# Supplementary material for: Patients’ experience of and participation in a stroke self-management programme, My Life After Stroke (MLAS): a multimethod study
Source: BMJ Open. 2022 Nov 15;12(11):e062700. doi: 10.1136/bmjopen-2022-062700 (PMC9668005; doi:10.1136/bmjopen-2022-062700)
Supplement: Supplementary data [file bmjopen-2022-062700supp001.pdf]

As part of the MLAS [illegible] GP [illegible] Practice Nurse [illegible] to take part in the **My Life After Stroke (MLAS)** programme.

If you decided **not** to take part, please can you tell us why?

____

____

____

____

____
