# Supplementary material for: Patients’ experience of and participation in a stroke self-management programme, My Life After Stroke (MLAS): a multimethod study
Source: BMJ Open. 2022 Nov 15;12(11):e062700. doi: 10.1136/bmjopen-2022-062700 (PMC9668005; doi:10.1136/bmjopen-2022-062700)
Supplement: Supplementary data [file bmjopen-2022-062700supp002.pdf]

Thank you for attending **Stroke Journey**.

Please tick (✓) **agree, disagree, don't know** or **does not apply** for each of the statements below.

	Agree	Disagree	Don't know	Does not apply
1. I felt like I could contribute to the session if I wanted to.				
2. I felt like I could participate in the session activities.				
3. The session included a discussion about my **stroke story**.				
4. I had a chance to think about where I am on my **journey** since my stroke.				
5. I know what a stroke is.				
6. I know how a stroke can affect me.				
7. I understand what **neuroplasticity** is.				
8. I have a **strategy** that can help me deal with the effects of my stroke.				
9. The session included a discussion about **feelings** after having a stroke.				

Please turn over.

Please tick ✓ **agree**, **disagree**, **don't know** or **does not apply** for each of the statements below.

	Agree	Disagree	Don't know	Does not apply
10. I am aware of the **barriers** to moving forward with my life after stroke.				
11. I had a chance to talk to other stroke survivors **without my carer**.				
12. I had a chance to complete the **'what will I do now?'** worksheet.				
13. I know what I am going to think about doing differently from today.				
14. I received my **'Stroke journey'** handbook.				

Please write your **comments** below:

Blatchford EG, *et al. BMJ Open* 2022; 12:e062700. doi: 10.1136/bmjopen-2022-062700

Thank you for attending **Managing health and wellbeing**.

Please tick (✓) **agree, disagree, don't know** or **does not apply** for each of the statements below.

	Agree	Disagree	Don't know	Does not apply
1. I felt like I could contribute to the session if I wanted to.				
2. I felt like I could participate in the session activities.				
3. The session included an activity on **risk factors for stroke**.				
4. I am aware of what my risks are for having another stroke.				
5. I know what I could do to help **reduce** my risk of having another stroke.				
6. I know what I could do to **stay healthy**.				
7. I understand the **pros and cons** to making change.				
8. I made an **action plan**.				
9. I am confident I can achieve my plan.				

Please turn over.

Improving primary care after stroke (IPCAS): A cluster randomised controlled trial
MLAS Patient Receipt Checklist – Group 2
IRAS Number: 233891
v.1.0 21-06-18

Please tick ✓ **agree**, **disagree**, **don't know** or **does not apply** for each of the statements below.

	Agree	Disagree	Don't know	Does not apply
10. There were opportunities to **ask questions** at the session.				
11. I was able to learn from other people in the group.				
12. I had a chance to complete the '**what will I do now?'** worksheet.				
13. I know what I am going to think about doing differently from today.				
14. I received my **'Managing health and wellbeing'** handbook.				

Please write your **comments** below:

Improving primary care after stroke (IPCAS): A cluster randomised controlled trial
MLAS Patient Receipt Checklist – Group 2
IRAS Number: 233891
v.1.0 21-06-18

Thank you for attending **Roadblocks**.

Please tick (✓) **agree, disagree, don't know** or **does not apply** for each of the statements below.

	Agree	Disagree	Don't know	Does not apply
1. I felt like I could contribute to the session if I wanted to.				
2. I felt like I could participate in the session activities.				
3. The group discussed the link between **thoughts, feelings, physical sensations, and behaviour**.				
4. The group discussed what problems or **'roadblocks'** people might face after a stroke.				
5. I had a chance to complete **'what are my roadblocks?'** worksheet.				
6. The session included a **problem-solving** activity.				
7. The facilitators invited me to share my problems and feelings.				
8. I am able to think about my own problems or roadblocks.				
9. I am able to think about how to solve or manage my own problems or roadblocks.				

Please turn over.

Improving primary care after stroke (IPCAS): A cluster randomised controlled trial
MLAS Patient Receipt Checklist – Group 3
IRAS Number: 233891
v.1.0 21-06-18

Please tick ✓ **agree**, **disagree**, **don't know** or **does not apply** for each of the statements below.

	Agree	Disagree	Don't know	Does not apply
10. There were opportunities to **ask questions** at the session.				
11. I was able to learn from other people in the group.				
12. I had a chance to complete the **'what will I do now?'** worksheet.				
13. I know what I am going to think about doing differently from today.				
14. I received my **'Roadblocks'** handbook.				

Please write your **comments** below:

Improving primary care after stroke (IPCAS): A cluster randomised controlled trial
MLAS Patient Receipt Checklist – Group 3
IRAS Number: 233891
v.1.0 21-06-18

Thank you for attending **'Moving forward on my journey'**.

Please tick (✓) **agree, disagree, don't know** or **does not apply** for each of the statements below.

	Agree	Disagree	Don't know	Does not apply
1. I felt like I could contribute to the session if I wanted to.				
2. I felt like I could participate in the session activities.				
3. I was able to think about how my relationships have changed since having had my stroke.				
4. The facilitators invited me to share my experiences of changing relationships.				
5. The session included a **problem-solving** activity.				
6. I had a chance to talk to other stroke survivors **without my carer**.				
7. I had a chance to think about where I am on my **journey** since my stroke.				
8. I am aware of how I can keep track of my **physical and mental health**.				
9. I know what I could do to **stay healthy**.				

Please turn over.

Please tick ✓ **agree**, **disagree**, **don't know** or **does not apply** for each of the statements below.

	Agree	Disagree	Don't know	Does not apply
10. There were opportunities to **ask questions** at the session.				
11. The group discussed ways to get **support or information**.				
12. I had a chance to complete the **'what will I do now?'** worksheet.				
13. I know what I am going to think about doing differently from today.				
14. I received my **'Moving forward on my journey'** handbook.				
15. I received my **'Directory'**.				

Please write your **comments** below:

Thank you for attending **Individual appointment 1**.

Please tick (✓) **agree, disagree, don't know** or **does not apply** for each of the statements below.

	Agree	Disagree	Don't know	Does not apply
1. The facilitator explained **'My life after stroke'** to me.				
2. The facilitator invited me to **ask questions** about 'My life after stroke'.				
3. I understand what **'My life after stroke'** is.				
4. I felt I could talk about what life was like **before and after** my stroke.				
5. The facilitator invited me to share any **problems I might face** in attending the course.				
6. I had the chance to talk about **ways to address** the problems I might face in attending the course.				
7. The facilitator invited my carer to come to my appointment.				
8. The facilitator invited my carer to **share experiences** if they wanted to.				

Please turn over.

Please write your **comments** below:

Thank you for attending **Individual appointment 2**.

Please tick (✓) **agree, disagree, don't know** or **does not apply** for each of the statements below.

	Agree	Disagree	Don't know	Does not apply
1. I was able to reflect on where I am on my **journey** after stroke.				
2. The facilitator invited me to talk about my experience of the **'My life after stroke'** course.				
3. The facilitator invited me to complete the **'Where am I on my journey?'** worksheet.				
4. I was able to **ask questions** if I wanted to.				
5. The facilitator and I talked about my **plans** after 'My life after stroke'.				
6. The facilitator checked if I had any questions.				
7. The appointment included **information about services** that I can access.				

Please turn over.

Please tick ✓ **agree**, **disagree**, **don't know** or **does not apply** for each of the statements below.

	Agree	Disagree	Don't know	Does not apply
8. My carer was invited to come to my appointment.				
9. The facilitator invited my carer to **share experiences** if they wanted to.				

Please write your **comments** below:

