# Supplementary material for: Patients’ experience of and participation in a stroke self-management programme, My Life After Stroke (MLAS): a multimethod study
Source: BMJ Open. 2022 Nov 15;12(11):e062700. doi: 10.1136/bmjopen-2022-062700 (PMC9668005; doi:10.1136/bmjopen-2022-062700)
Supplement: Supplementary data [file bmjopen-2022-062700supp003.pdf]

Improving Primary Care After Stroke: A randomised controlled trial to evaluate a new model of care for stroke survivors living in the community

Proposed survivor Topic Guide for semi-structured interviews

Interview with a stroke survivor – Intervention Arm

Thank you for agreeing to let me come and talk with you. We're interested to hear from people who've taken part in the IPCAS trial, and your experiences of stroke care so far.

Confidentiality & anonymity. Tape recording. Stopping / Pausing. Anything you don't want to talk about. I'm not a clinician. Any questions?

Is there anything you'd like me to know before we start that would make things easier for you? For instance, do you want me to sit one side or another? Do you sometimes find it difficult to find the right word? Or to understand what someone's saying? Is there anything I can do to make things better for you?

Do you have any questions?
Provide brief outline / reminder of what the study was about

Review at GP Surgery

Can you tell me about your experience of the stroke review you attended at your GP surgery?
(*prompts: date of review, person who did the review, checklist*)

Can you tell me about what happened at the review?
(*prompts: checklist discussion, action plan, blood pressure, any recommendations regarding social care/support services*)

What were your expectations of the stroke review?
(prompts: were they met/not met? How?)

Overall, what did you think about the checklist used for your review?
For example, was it helpful/useful? Why/why not?
(*prompts: did it help you identify and discuss needs you may have, were you referred on, were you given information about services available to you*)

Overall, what did you think about the review?
For example, was it helpful/useful? Why/why not?
(*prompts: were you referred on, did you attend MLAS, were you given information about services available to you*)

How did this review meet your needs in relation to your stroke?
Is there anything else you wanted to share about your stroke review today?

If no review – do you think a review of your care needs at your GP Surgery would have been helpful/useful? Why/why not?

Blatchford EG, *et al. BMJ Open* 2022; 12:e062700. doi: 10.1136/bmjopen-2022-062700

MLAS self-management course

Can you tell me a little bit about your experience of the MLAS self-management course?
(prompts: Did you attend all sessions?; What parts of MLAS did you like the most/least liked? Why?)

How did you hear about MLAS?
(*prompts: what information did you get?*)

How did MLAS meet your needs in relation to your stroke?

Overall, what did you think about the MLAS course?
(*prompts: did it help you to feel more confident/independent/empowered to manage you stroke related needs*)

Was it helpful/useful? If yes, how? If no, why not?

Is there anything else you wanted to share about participating in MLAS today?

If participant did not attend MLAS:
Were you told about MLAS at the review? If yes: can you please tell me a bit about your reasons for not attending MLAS (prompts as below).
Did you choose not to attend, or were there other reasons (e.g. logistic, travel, cost, time) that prevented you from attending?

Direct Point of Contact

Tell me about how you used the direct point of contact service that you were told about at your stroke review.

If participant did not use the service, explore: what were your reasons for not using the direct point of contact?
(prompts: no information given, did not want to bother the Practice staff, not feel it was worth it, couldn't get through on the telephone)

Would you have liked to be able to contact someone at your GP surgery about your stroke needs?

How useful do you think it would have been to have a direct point of contact/somebody specific to call? Explore reasons why/why not.

What prompted you to call the direct point of contact service?

How much did you use the direct point of contact service?

How useful was this direct point of contact service?
(prompts: What kind of information/advice/support did you receive?)

What do you think about the response you received?
(prompts: Called back in a timely manner, extent to which issues/questions/needs were addressed)

Overall

Since you entered the IPCAS trial, what changes have you noticed in how your GP surgery deals with your needs/concerns/issues?
(*prompts: easier/harder to contact, discuss problems, more/less helpful service*)

What are your views about the care that you received as a whole?
(prompts: Thoughts on the information/advice given throughout? What did you learn (if anything)? How are you using your learning?)

Is there anything you think needs to change about your stroke care? If so, what are these changes, and why?

Is there anything else you would like to discuss relating to the care you received as part of the IPCAS trial?

Thank you very much. These are all questions I had. Is there anything you would like to ask?

Thank you very much for your time.

Blatchford EG, *et al. BMJ Open* 2022; 12:e062700. doi: 10.1136/bmjopen-2022-062700
