# Supplementary material for: Patients’ experience of and participation in a stroke self-management programme, My Life After Stroke (MLAS): a multimethod study
Source: BMJ Open. 2022 Nov 15;12(11):e062700. doi: 10.1136/bmjopen-2022-062700 (PMC9668005; doi:10.1136/bmjopen-2022-062700)
Supplement: Author's manuscript [file bmjopen-2022-062700.draft_revisions.pdf]

BMJ Open is committed to open peer review. As part of this commitment we make the peer review history of every article we publish publicly available.

When an article is published we post the peer reviewers' comments and the authors' responses online. We also post the versions of the paper that were used during peer review. These are the versions that the peer review comments apply to.

The versions of the paper that follow are the versions that were submitted during the peer review process. They are not the versions of record or the final published versions. They should not be cited or distributed as the published version of this manuscript.

BMJ Open is an open access journal and the full, final, typeset and author-corrected version of record of the manuscript is available on our site with no access controls, subscription charges or pay-per-view fees (http://bmjopen.bmj.com).

If you have any questions on BMJ Open's open peer review process please email info.bmjopen@bmj.com

BMJ Open

Patient experience of and participation in a stroke self-management programme: My Life After Stroke (MLAS)

Journal:	*BMJ Open*
Manuscript ID	bmjopen-2022-062700
Article Type:	Original research
Date Submitted by the Author:	09-Mar-2022

Complete List of Authors:	Blatchford, Emily; University of Cambridge Primary Care Unit, Primary Care Unit<br>Aquino, Maria; NIHR Applied Research Collaboration North East and North Cumbria, Population Health Sciences Institute<br>Grant, Julie; University of Cambridge Primary Care Unit, Primary Care Unit<br>Johnson, Vicki; Univ Hosp Leicester NHS Trust, Leicester Diabetees Centre<br>Mullis, Ricky; University of Cambridge Primary Care Unit, Primary Care Unit<br>Lim, Lisa; University of Cambridge Primary Care Unit, Primary Care Unit<br>Mant, Jonathan; University of Cambridge Primary Care Unit, General Practice and Primary Care Research Unit

Keywords:	Stroke < NEUROLOGY, PRIMARY CARE, PUBLIC HEALTH

SCHOLARONE™
Manuscripts

BMJ

I, the Submitting Author has the right to grant and does grant on behalf of all authors of the Work (as defined in the below author licence), an exclusive licence and/or a non-exclusive licence for contributions from authors who are: i) UK Crown employees; ii) where BMJ has agreed a CC-BY licence shall apply, and/or iii) in accordance with the terms applicable for US Federal Government officers or employees acting as part of their official duties; on a worldwide, perpetual, irrevocable, royalty-free basis to BMJ Publishing Group Ltd ("BMJ") its licensees and where the relevant Journal is co-owned by BMJ to the co-owners of the Journal, to publish the Work in this journal and any other BMJ products and to exploit all rights, as set out in our licence.

The Submitting Author accepts and understands that any supply made under these terms is made by BMJ to the Submitting Author unless you are acting as an employee on behalf of your employer or a postgraduate student of an affiliated institution which is paying any applicable article publishing charge ("APC") for Open Access articles. Where the Submitting Author wishes to make the Work available on an Open Access basis (and intends to pay the relevant APC), the terms of reuse of such Open Access shall be governed by a Creative Commons licence – details of these licences and which Creative Commons licence will apply to this Work are set out in our licence referred to above.

Other than as permitted in any relevant BMJ Author's Self Archiving Policies, I confirm this Work has not been accepted for publication elsewhere, is not being considered for publication elsewhere and does not duplicate material already published. I confirm all authors consent to publication of this Work and authorise the granting of this licence.

For peer review only

Patient experience of and participation in a stroke self-management programme: My Life After Stroke (MLAS)

Authors:

Emily Grace Blatchford, MA, BSc[1]
Maria Raisa Jessica (Ryc) Aquino, PhD [1,2]
Julie Grant, BSc [1]
Vicki Johnson, BSc [3]
Ricky Mullis, PhD[1]
Lisa Lim, MB BChir[1]
Jonathan Mant, MD[1]

1 Primary Care Unit, Department of Public Health and Primary Care, University of Cambridge, UK

2 NIHR Applied Research Collaboration North East & North Cumbria, Population Health Sciences Institute, Faculty of Medical Sciences, Newcastle University, UK

3 Leicester Diabetes Centre, University Hospitals Leicester NHS Trust, UK

Corresponding author:

Emily Grace Blatchford
egb39@cam.ac.uk
Primary Care Unit, Department of Public Health and Primary Care, University of Cambridge, UK

For peer review only

Abstract

Objective

A self-management programme, My Life After Stroke (MLAS), was developed to support stroke survivors. This evaluation reports patient experience.

Design

Mixed methods, involving interviews and questionnaires

Setting

23 general practices in the intervention arm of a cluster randomised controlled trial in East of England and East Midlands, UK

Participants

People on the stroke registers of participating general practices were invited to attend an MLAS programme.

Interventions

MLAS comprises one-to-one and group-based sessions to promote independence, confidence, and hope.

Primary and secondary outcome measures

The primary outcome was uptake of the programme. Participants who declined MLAS were sent a questionnaire to ascertain why. Attendees of four programmes completed evaluation forms. Attendees and Non-attendees of MLAS were interviewed. Ad-hoc email conversations with the lead author were reviewed. Thematic analysis was used for qualitative data.

Results

141/420 (34%) of participants (mean age 71) attended an MLAS programme and 103 (73%) completed one. 64/228 (28%) participants who declined MLAS gave reasons as: good recovery; ongoing health issues; logistical issues; inappropriate. Nearly all attendees who completed questionnaires felt that process criteria such as talking about their stroke and outcomes such as developing a strong understanding of stroke had been achieved.

Conclusions

MLAS was a positive experience for participants but many stroke survivors did not feel it was appropriate for them. Participation in self-management programmes after stroke might be improved by offering them sooner after the stroke and providing a range of delivery options beyond group-based, face-to-face learning.

For peer review only

Article Summary

Strengths and Limitations

- The self-management programme used in this study was developed using extensive patient and public involvement; the programme has been previously demonstrated to be feasible to deliver in this setting
- The study population is people on general practice stroke registers rather than those recruited from specialist stroke services, so better reflects the experience of a self-management programme of longer-term community dwelling stroke survivors
- The study does not provide data on effectiveness of this self-management programme, but on uptake and patient experience
- The mixed methods approach allowed us to understand the reasons that people do not take part in these programmes.

For peer review only

Introduction

Background

There is a lack of support for stroke survivors living in the community[1,2,3]. Stroke survivors report a sense of abandonment and a need for social, psychological, and educational support[4,5,6,7]. Psychological issues are associated with higher rates of mortality, long-term disability, hospital readmissions, suicide and higher utilisation of outpatient services[8,9,10]. The need for development and evaluation of different models of care to address long-term needs of stroke survivors in primary care is well recognised[11], yet such models remain scarce[12]. Adjusting to life after stroke continues after formal rehabilitation has finished[13,14]. The evidence-base for the role of self-management programmes is growing[15]: such approaches have been successfully applied to long-term conditions such as diabetes[16,17] and can reduce pressure on social and healthcare services[18] by allowing participants to take an active role in their own condition, making informed choices whilst adopting behaviour and psychological changes[15,19,20,21].

Self-management programmes have been used to support stroke survivors during their aftercare. Programmes such as the New Start[22,23] intervention, the Australia-based Stroke Self-Management Programme (SSMP)[24], Bridges[25], and the Canadian Moving On after STroke (MOST)[26] include processes such as increasing contact time, identifying needs, exploring social networking, problem-solving, goal setting, forming action plans, providing stroke-specific information, using trained healthcare professionals and increasing exercise. MOST-Telehealth Remote[27] took self-management virtually using video conferencing to remotely connect facilitators with participants further afield.

As part of a new primary care-based model of care, Improving Primary Care After Stroke (IPCAS)[28], a novel self-management programme, My Life After Stroke (MLAS) was developed using the MRC framework and extensive patient and public involvement (PPI), to target the long-term needs of stroke survivors[29]. Areas covered include social wellbeing, community re-integration, emotional support, physical needs, activities of daily living and prevention of future strokes, informed by self-efficacy and cognitive behavioural therapy (CBT). This intervention is one of few that focus on stroke survivor self-reported needs. MLAS was subsequently found to be feasible to deliver[30], and so was included as one of the components of IPCAS that was evaluated in a randomised controlled trial[28].

Objective

While it was not possible to directly evaluate any of the individual components within the IPCAS model of care using the randomised design, we wanted to better understand the potential role of self-management programmes such as MLAS. This paper therefore focuses on patient experience of and participation in MLAS as part of a larger process evaluation[31].

For peer review only

Methods

MLAS

MLAS programmes (n=24) were held between September 2018 and January 2020 across six counties in England: Norfolk (n=8), Suffolk (n=4), Northamptonshire (n=4), Cambridgeshire (n=5), Leicestershire (n=2) and Essex (n=1). Programmes lasted nine weeks with over eleven hours' contact time[29]. There is an individual appointment with a facilitator at the start of the programme, and a second one at the end. In between individual appointments, there are four group sessions (2.5 hours each) covering different topics. The completion criterion states participants must attend both individual appointments, group session-1 and a minimum of two other group sessions. Individual appointments (face-to-face or telephone) consist of a discussion to build rapport and explore the impact of their stroke before and after MLAS. Group sessions were interactive, consisted of social support and signposting to local services and topics including managing physical and emotional health, warning signs, and risks of stroke. All sessions aimed to build confidence, independence and hope through problem-solving, Cognitive Behavioural Therapy (CBT)-based approaches, and opportunities to share personal experiences. The same two facilitators ran all six sessions of a programme (where possible) and were trained and mentored in behaviours such as being non-judgemental, not giving specific advice, effective communication and active listening skills, and how to use the MLAS facilitator curriculum and resources to deliver the intervention as intended. Participants were provided with refreshments, programme handbooks and each session began with a recap of previous sessions.

Recruitment

Participants were in the intervention arm of the IPCAS cluster randomised trial[28]. People were eligible to take part in IPCAS if they were on the stroke register of a participating general practice, were 18 years or older and able to provide written informed consent, with or without the help of a carer. They were invited to attend a programme whilst at a stroke review led by a health care professional at their GP surgery. Invitees were given the study team's contact details and the lead author (EB) contacted all participants two weeks post-invitation by telephone or email. Those expressing interest were booked onto a programme in an accessible (e.g., parking, wheelchair-friendly and accessible facilities) community venue near their general practice surgery. Pre-arranged and funded return-transport was available. Carers and/or significant others were encouraged to attend with participants.

Facilitators

Facilitators (n=23) were trained by the intervention developers during 2018/19[29]. Facilitators' backgrounds included nursing, psychology, and those with specific interest in stroke.

Methods of data collection

Qualitative and quantitative data were collected in order to answer the questions below.

For peer review only - http://bmjopen.bmj.com/site/about/guidelines.xhtml

Why did participants decline MLAS?

Participants were sent a questionnaire 6-months post-randomisation which included a question asking if they hadn't attended MLAS, why that was (see appendix A). In some areas, there were delays with set-up of MLAS which meant that participants' received the questionnaire before their invitation to attend the programme were unable to provide a response to this question.

What did participants experience when attending MLAS?

All attendees from a purposefully selected sample of programmes(n=4) received an evaluation form to complete at the end of each MLAS session which explored what happened during the session and what they took away from it. Programme selection criteria included facilitators leading programmes and geographical location. We also aimed to include participants that were not participating in other elements of the wider process evaluation (e.g., interviews, see 2.4.3) to offer individuals opportunities to share their experiences, and at the same time, minimise participant burden. Responses were on a Likert scale and included a comments box (see appendix B).

What are participant views on MLAS?

Participants were invited to take part in semi-structured telephone interviews. Potential participants were selected to ensure that people who completed a programme, started a programme but did not complete it, declined to take part, or agreed to take part but did not attend were all included. Interviews were performed by JG and followed a topic guide used as a prompt for open-ended discussion suitable for differing levels of cognitive function. Topics covered experience and participation, informed by the Behaviour Change Consortium framework[32].

What feedback was given after MLAS was delivered?

Informal general feedback was given to the lead author (EB) from both participants and facilitators via email post-attendance.

Analysis

The interview transcripts were coded and analysed inductively by two researchers (EB,JG) using thematic analysis[33,34,35,36]. Transcripts were repeatedly read and coded using NVivo software to develop themes and sub-themes. Two coders (EB, JG) individually coded the interview data for phrases related to MLAS, including terms of enjoyment, learning etc., which were summarised and categories were formed. The coders met to compare and revise their codes and develop themes. Disagreements were resolved through discussions with a third member of the research team (RA). The qualitative data from questionnaires and ad-hoc feedback was analysed by the lead author (EB) using thematic analysis as above. Patient receipt questionnaires were analysed for process and outcome data and simple descriptive statistics were used for the quantitative data. A two sample *t* statistic was used to compare if there was a difference between those who attended and did not attend, in relation to the distance from individuals' home to the programme venue.

For peer review only

Patient and Public Involvement

We actively engaged PPI in the development of the Managing Life After Stroke self-management programme through lay membership of our Programme Steering Committee and our Intervention Development Group, a lay representative co-applicant, active engagement with The Stroke Association, and in several meetings with stroke survivor and carer support groups in the community.

Consultation meetings took place with stroke survivors in April 2016 (2 in Leicester, 1 in Cambridge - 20 people per group) to seek input on content of the self-management intervention and its format.

The topic guides for our interviews were pilot tested with PPI members via email, telephone calls and a mock interview.

Results

522 stroke survivors were randomised to the intervention arm. Fifty-nine did not attend the stroke review, 42 withdrew from the trial and 2 died. As a result, 420 individuals were invited to attend an MLAS programme. 192 (46%) participants who were invited to MLAS expressed interest in attending. Of those expressing interest, 141 (34%) attended, and 103 (73%) completed a programme.

Participant characteristics are shown in Table 1. 94% (133/141) of participants attended a programme more than a year after their stroke. There were no differences between those who attended and did not attend. 70% (99/141) of participants attended a programme within 2-miles from their residence. The mean distance from the programme venue to the stroke survivor's home was not significantly different between those who did and did not attend the programme (*p-value* 0.26).

Table 1
Characteristics of stroke survivors by whether or not they attended the MLAS programme

	**Attended (n=141)**	**Expressed Interest but Did Not Attend (n=51)**	**Did not wish to attend (n=228)**
**Gender**			
Male	62.4% (n=88)	62.7% (n=32)	66.7% (n=152)
Female	37.6% (n=53)	37.3% (n=19)	33.3% (n=76)
**Ethnicity**			
White	97.9% (n=138)	100.0% (n=51)	96.5% (n=220)
Other	2.1% (n=3)	0.0% (n=0)	3.5% (n=8)
**Age**			
Mean Age At Baseline	71.2	70.9	69.7
**Time Since Stroke (tss)**			
Mean tss to starting MLAS	8.2 years		

For peer review only

Distance to MLAS Venue			
Mean miles from residence to MLAS venue	2.4 Miles	3.5 Miles	2.8 Miles

Why did participants decline MLAS?

Sixty-four participants gave multiple reasons for declining MLAS which were grouped into four main themes: Good recovery, Ongoing health issues, Logistical issues, and Not appropriate (Figure 2).

Good Recovery

28 people felt they made a good or full recovery, experienced no lasting side effects, and their stroke was a long time ago; therefore no longer needed further support.

Ongoing Health Issues

13 people had ongoing health or medical issues such as age-related concerns, cancer treatments, short-term pre-planned hospital visits or minor illnesses.

Logistical issues

9 people were unable to attend due to other commitments such as carer duties, the programme being too time consuming, inconvenient days/times and travel issues.

Not Appropriate

22 people did not feel the programme was appropriate for them, including disliking group sessions, lacking confidence, uncertainty about the benefits of attending, and concerns about the programme being too distressing.

The most common reason given by people who had indicated they would like to attend but subsequently did not was that it was being held at an inconvenient time. Views expressed on self-management programmes in general by non-attenders were positive; they thought they would be useful, tailored to individual needs, good for social aspects, and learning.

Interviews were conducted with a small sample of participants who had been invited to MLAS but declined (n=3) or expressed interest but did not attend (n=2). Reasons matched those in Figure 2, with additional ones including: the programme having no connection to their stroke, previously attending similar programmes, being in full-time employment, and moving away.

What did participants experience when attending MLAS?

Figures 3 and 4 show the extent to which participants agreed that the programmes were carried out as intended. 18 participants were invited to complete evaluation forms. There were 54 process criteria which included learning about MLAS, talking about one's stroke, asking questions, and forming discussions. All participants who responded felt these were met with the exception of three criteria, where in each case only 1 or 2 participants felt otherwise. Similarly, there were 20 outcome criteria which were largely concerned with different aspects of knowledge about stroke.

For peer review only

There were only two criteria which a responder felt were not met relating to confidence in building action plans and acting upon them.

Free text collected from these forms (n=14) was positive and showed participants had "interesting discussions", "thoroughly enjoyed the course", found it "useful" and enjoyed "meeting fellow members and hearing their experiences". One participant said "I have really pushed myself to get past my roadblock and have started to use my quadstick to move around short distances (with somebody close by). I am sure I would not have attempted this without attending these little meetings".

What are participants' views on MLAS?

Interviews were conducted with a small sample of participants who had been invited, attended and/or completed MLAS (n=12). Four themes were identified: Programme characteristics influence participation, Programme content is important, Perceived benefits of attendance, and Personal circumstances affect participation.

Programme Characteristics Influence Participation

Participants (n=4) spoke about the logistics of programmes, referring to preferred days of the week, time of day, location (closer to home the better) and transport (taxis benefited participants with mobility issues). Participants (n=5) also discussed the structure of group sessions (some enjoyed groups and others did not), size of groups (smaller groups preferred) and length of sessions (2-hours was long enough). One participant said "I think six [participants] was quite nice" [female, 68]. Another participant said: "I can't cope when there's lots of people in the room and too much information is coming in…too many people's voices" [female, 56].

Programme Content Is important

Participants (n=6) reported that programme content should meet attendees' individual needs. One participant explained, "not everyone's difficulties are so obvious" [female, 81] as participants had their stroke at different times in their lives resulting in various after-effects. Another participant explained, "I struggle a lot with my memory...I can forget, you know, talking to you, I'd forget your name at the end" [female, 58]. By remaining flexible, participants were able to complete handbooks at their own pace without feeling pressured.

Perceived Benefits of Attendance

Participants (n=6) reported a change in self-awareness, with a less negative mind-set. They also (n=4) perceived change in their behaviour, including increasing exercise and engaging in hobbies; one participant said, "I was sitting there thinking there isn't anything I can do, but then I remembered I'd got this big purple exercise ball. So, it actually made me get it out, and I've got a new sewing machine...so now that's made me buzz, because now I'm back into patchwork and quilting so I think right, I'll join a patchwork and quilting group. So that was a very positive thing that came out of it. Creatively, I'm just buzzing" [female, 56]. Thirdly, participants (n=10) reported that the programme increased their knowledge of stroke. Finally, participants (n=11) considered attendance to be a positive experience, gaining social support and engaging in altruism; a participant said "I found it so helpful just listening

For peer review only

to other people's stories and feeling able to talk about my own feelings so that was very, very helpful" [female, 78].

Personal Circumstances Affect Participation

Participants (n=7) said individual circumstances affected their participation, including having existing health comorbidities and how far into recovery they were. One participant said "with my eyes it is quite difficult to do all the things that I used to do...I can't use my legs and now I can't use my eyes either" [female, 56] another participant said "it's [been] five years since I had the stroke, but most people at the group, it was a longer time since they'd had their strokes…I know this is a new project, but I would have found it helpful to have had this course much nearer to the stroke" [female, 73].

What feedback was given after MLAS was delivered?

Informal feedback was received from participants (n=2) and facilitators (n=10) via emails after attending an MLAS programme and grouped into three themes.

Participants had a positive experience

Participants expressed positive experiences and found the programme useful. One said he was "sceptical at first about joining the course and felt it would be a waste of time" but has "learnt so much and is glad he attended" [70, male]. Another mentioned it was "good to speak with other stroke survivors" and found problem solving "most useful when it came to putting it into real life" [78, female]. A facilitator said that one participant didn't realise how angry he was, but by the second group session "felt like a weight had been lifted" [Northamptonshire programme]. Facilitators recognised participants were positive when completing the programmes as they had learnt from each other and shared personal experiences.

Programme more beneficial closer to time of stroke onset

Participants' strokes were often not recent, one stated that the programme "would be more useful when you come home from hospital". Facilitators explained how participants struggled to identify areas of difficulty as they no longer fitted the stereotypical stroke survivor.

Ongoing health issues

Facilitators expressed the view that many of participants had ongoing health comorbidities which were often more pronounced than the consequences of their stroke.

Discussion and Conclusion

Key Findings

Participants who attended MLAS had a positive experience and felt that the programmes achieved what was intended. However, over half of those invited declined to attend due to good recovery, ongoing health issues, logistical issues, or not feeling the programme was appropriate for them.

For peer review only

Strengths and Limitations

More participants (n=141) took part in MLAS compared to evaluations of other self-management programmes for stroke[22,23,24,26,27,37]. Using a mixed methods approach helped to identify reasons for low participation, and explore why those who had attended MLAS felt it was a success. Furthermore, consistent themes were identified from the different sources of data. All qualitative data were double coded, with a third experienced analyst resolving disagreements. Only 32% of people who declined to take part in a programme gave an explanation as to why this was, but these data were supplemented by five interviews with those who did not attend. Only a small sample of attendees received an evaluation form (13%) from four programmes (17%), to minimise participant burden. The use of emails provided a perspective from the facilitators, which complemented that of participants. However, there was a lack of diversity within the sample, participants being primarily white, retired, and more than a year post stroke. Participants may not have been representative of the whole population of stroke survivors as they were all taking part in a randomised controlled trial.

Comparison with existing literature

There is some evidence from randomised controlled trials that self-management programmes may improve quality of life and self-efficacy[15]. With regard to patient experience and participation, there have been insights from before and after studies and randomised control trials (RCT). The New Start intervention[37], which has similarities to MLAS, is a self-management programme for patients within the first six months of their stroke which is being tested in an ongoing RCT[22,23]. The SSMP intervention[24] was part of an RCT that aimed to recruit participants of similar age to MLAS >3months post stroke to an eight week programme; 70% of attendees were >12months post stroke, which is similar to MLAS. The MOST intervention[26] compared a new self-management programme with 34-hours of group contact time over eight weeks to a standard education programme >3months post stroke. It involved three times as much contact time as MLAS using similar sized groups and ages. MLAS does appear to have a slightly lower uptake than these programmes but a higher completion rate. The SSMP intervention[24] had 53% (27/48) attendance compared to 34% (141/420) in MLAS, with 52% (25/48) completing >50% of sessions compared to MLAS which had 90.1% of participants complete more than 50% of sessions. This could be due to participants receiving dates in advance and having transport provided. The MOST intervention[26] had a small 60% (18/30) participant recruitment and only 22% (4/18) attended all of the sessions compared to 53.2% for MLAS. Qualitative feedback from participants of these other programmes was positive, as it was for MLAS. Reasons for non-attendance of the SSMP course (time constraints, lack of interest and inability to attend e.g., because of transportation issues) were similar to MLAS. A follow up study to MOST[26], MOST-TR[27] involved video conferencing with 36-hours of online-contact time over a nine week period with participants 3-18-months post stroke. Participants valued access to this program without the need to travel. A review[15] of the effectiveness of self-management programmes for community living adults with stroke identified 14 RCTs conducted across eight countries between 2000-2015 with 1863 participants. All programmes were community, home or out-patient based and lasted a duration of four-weeks to six-months. There was some evidence of improved quality of life (the

For peer review only

primary outcome). Secondary outcome measures showed positive improvements in self-efficacy, self-esteem, emotional stability, anxiety and depression and mood.

Interpretation

Given that there is some evidence that self-management programmes can improve quality of life in stroke survivors,[15] it is important to understand why people do or do not attend such programmes. While people who attended the MLAS programme found it helpful, many people in our sample did not feel that the programme was suitable for them. Some people felt that it had been too long since their stroke had passed to be useful to attend, though other programmes that recruited participants earlier after their stroke also had poor uptake[24,26,27]. It is a feature of primary care that most people on stroke registers will not have had a recent stroke, and so this raises the question as to whether self-management programmes might be more useful if accessed from specialist secondary care services or offered at the point of discharge or engagement with early supported discharge (ESD). Other potential participants felt they had too many ongoing health problems to attend, highlighting the problem of providing single-condition programmes for people with multimorbidity[38,39]. For those who do wish to attend a programme, it needs to be adaptable to patient needs and wishes. For example, it has been shown to be feasible to provide virtual sessions which can overcome attendance barriers[40]. Tailoring programmes might also address concerns of people who do not like group work. It is possible the decision to take part in an MLAS programme was influenced by how it was introduced to the stroke survivor by the healthcare professional. It may be that uptake could be improved by focussing on this aspect. Before widespread adoption of self-management programmes such as MLAS, further evidence is required on optimal timing, content, and mode of delivery[15].

Conclusion

The mixed-methods and multi-perspective nature of this analysis has provided insights into patient experience of and participation in a stroke self-management programme, MLAS. This intervention, which focuses on addressing patient-self-reported needs, was found to provide a positive experience for participants. Although many stroke survivors who declined participation did not feel it was appropriate for them, it might have a role as part of a menu of optional services.

Practical Implications

This study suggests that there should not be an expectation that all stroke survivors would want to take part in a self-management programme such as MLAS. There was feedback that the programme would be more valuable if offered sooner after the stroke. This could be achieved by having referring routes from specialist services rather than from primary care, though uptake is not necessarily higher from these settings. Some potential participants did not like the idea of group work, and others found it logistically difficult to attend. Setting up on-line sessions (whether individual or group based) would be a way of addressing such concerns.

For peer review only

Figure Legend

Figure 1 - Study flow chart for those invited, attended and declined MLAS
Figure 2 - Venn diagram of themes for declining the self-management programme, MLAS
Figure 3 - Patient evaluation forms, Process questions. n= number of participants attending the session
Figure 4 - Patient evaluation forms; Outcome questions. n= number of participants attending the session – Individual appointment 2 had no outcome questions

Acknowledgements

We are thankful for the IPCAS administration team members who included Lizzie Kreit, Victoria Theobald and Alex Newbrook. With thanks to the trainers from Leicester and the MLAS facilitators, who without them, the programmes would not have been delivered in the community venues across England. Thank you to the stroke survivors participating in the study. Thank you to Bundy Mackintosh, patient adviser.

Funding

This study is funded by the National Institute for Health Research's Programme Grant for Applied Research titled 'Developing primary care services for stroke survivors' reference PTC-RP-PG-0213-20001. The views expressed are those of the authors and not necessarily those of the NIHR or the Department of Health and Social Care. Jonathan Mant is an NIHR Senior Investigator.

Declaration of competing interests

Vicki Johnson declares that she is employed by the University Hospitals of Leicester NHS Trust within the Leicester Diabetes Centre (LDC), which receives not-for-profit income for DESMOND, a suite of self-management programmes for which LDC holds the Intellectual Property Rights for. UHL also receives various grants to pay staff within LDC to carry out and implement various studies and self-management programmes. The Intellectual Property for MLAS is held by University of Leicester on behalf of UHL. All other authors declare that they have no competing interests.

Author Statement

- Emily Grace Blatchford: conceptualization; methodology, investigation, data curation; writing- original draft; visualisation.
- Maria Raisa Jessica Aquino: conceptualization; methodology; writing – review & editing; project administration; supervision.
- Julie Grant: investigation; data curation; writing - review & editing.
- Vicki Johnson: conceptualization; resources; writing – review & editing.
- Ricky Mullis: conceptualization; writing – review & editing; supervision; project administration; funding acquisition.
- Lisa Lim: conceptualization; writing – review & editing

For peer review only

- Jonathan Mant: conceptualization; writing – review & editing; supervision; funding acquisition.

Trial Registration

IPCAS ClinicalTrials.gov identifier (NCT number): ***NCT03353519***

Data Statement

Data are available upon reasonable request. The data may be available on request from the authors, subject to appropriate permissions: please email the PI Jonathan Mant (jm677@medschl.cam.ac.uk) for details.

Ethics Statement

It does involve human participants and has been approved by an Ethics Committee, Yorkshire & The Humber-Bradford Leeds NHS Research Ethics Committee (17/YH/0441)

For peer review only

References

1. National Audit Office (2010). Progress in improving stroke care: Department of Health, HC 291, Report by Comptroller and Auditor General, Session 2009–10. Department of Health: Progress in improving stroke care - National Audit Office (NAO) Report

2. McKevitt, C., Fudge, N., Redfern, J., Sheldenkar, A., Crichton, S., Rudd, A. R., ... & Wolfe, C. D. (2011). Self-reported long-term needs after stroke. *Stroke*, *42*(5), 1398-1403. DOI: 10.1161/STROKEAHA.110.598839

3. Pindus, D. M., Mullis, R., Lim, L., Wellwood, I., Rundell, A. V., Abd Aziz, N. A., & Mant, J. (2018) 'Stroke survivors' and informal caregivers' experiences of primary care and community healthcare services – A systematic review and meta-ethnography', *Plos One*, 13(4). doi: 10.1371/journal.pone.0192533.

4. Hafsteinsdóttir, T. B., Vergunst, M., Lindeman, E., & Schuurmans, M. (2011) 'Educational needs of patients with a stroke and their caregivers: A systematic review of the literature', *Patient education and counselling*. Elsevier, *85*(1), pp. 14–25. DOI: 10.1016/j.pec.2010.07.046

5. Taylor, S. J. C. *et al.* (2014) 'A rapid synthesis of the evidence on interventions supporting self-management for people with long-term conditions: PRISMS – Practical systematic RevIew of Self-Management Support for long-term conditions', *Health Services and Delivery Research*, 2(53), pp. 1–580. DOI: 10.1016/j.pec.2010.07.046

6. White, C. L., Barrientos, R. and Dunn, K. (2014) 'Dimensions of Uncertainty After Stroke', *Journal of Neuroscience Nursing*, 46(4), pp. 233–240. DOI: 10.1016/j.pec.2010.07.046

7. Chun, H.-Y. Y. *et al.* (2018) 'Anxiety After Stroke: The Importance of Subtyping', *Stroke; a journal of cerebral circulation*, 49(3), pp. 556–564. DOI: 10.1016/j.pec.2010.07.046

8. House, A. O. *et al.* (2001) 'Interventions for emotionalism after stroke', *The Cochrane Database of Systematic Reviews (Protocol)*. John Wiley & Sons, Ltd., (1). doi: 10.1002/14651858.cd003690.

9. Pohjasvaara, T. *et al.* (2001) 'Depression is an independent predictor of poor long-term functional outcome post-stroke', *European Journal of Neurology*, 8(4), pp. 315–319. DOI: 10.1016/j.pec.2010.07.046

10. Williams, L. S. (2005) 'Depression and Stroke: Cause or Consequence?', *Seminars in Neurology*, 25(04), pp. 396–409. DOI: 10.1016/j.pec.2010.07.046

11. Williams, L. S. and Rudd, A. G. (2010) 'Advances in health policy and outcomes 2009', *Stroke; a journal of cerebral circulation*, 41(2), pp. E77–80. DOI:

For peer review only

10.1016/j.pec.2010.07.046

12. Lennon, S., McKenna, S. and Jones, F. (2013) 'Self-management programmes for people post stroke: a systematic review', *Clinical Rehabilitation*, 27(10), pp. 867–878. DOI: 10.1016/j.pec.2010.07.046

13. Cott, C. A., Wiles, R., Devitt, R. (2007) Continuity, transition and participation: preparing clients for life in the community post stroke. *Disability and Rehabilitation* 29(20-21):1566-74. DOI: 10.1016/j.pec.2010.07.046

14. Pound, P., Gompertz, P., Ebrahim, S. (1998) A patient-centred study of the consequences of stroke. *Clinical Rehabilitation* 12:255-64. DOI: 10.1016/j.pec.2010.07.046

15. Fryer, C.E., Luker, J.A., McDonnell, M.N., Hillier, S.L. (2016) Self-management programmes for quality of life in people with stroke. *Cochrane Database of Systematic Reviews*. Issue 8. Art. No.: CD010442. DOI: 10.1002/14651858.CD010442.pub2.

16. Skinner, T. C. et al. (2006) 'Diabetes education and self-management for ongoing and newly diagnosed (DESMOND): Process modelling of pilot study', Patient Education and Counselling, 64(1-3), pp. 369–377. DOI: 10.1016/j.pec.2010.07.046

17. Davies, M. J. et al. (2008) 'Effectiveness of the diabetes education and self-management for ongoing and newly diagnosed (DESMOND) programme for people with newly diagnosed type 2 diabetes: cluster randomised controlled trial', Brit Med J , 336(7642), pp. 491–495. DOI: 10.1016/j.pec.2010.07.046

18. de Silva, D. (2011) Evidence: Helping people help themselves: A review of the evidence considering whether it is worthwhile to support self-management. The Evidence Centre.

19. Lorig, K.R., Mazonson, P.D., Holman, H.R.. (1993) Evidence suggesting that health education for self-management in patients with chronic arthritis has sustained health benefits while reducing health care costs. *Arthritis & Rheumatism* 36(4):439-46. DOI: 10.1016/j.pec.2010.07.046

20. Jones, F., Riazi, A. (2011) Self-efficacy and self-management after stroke: a systematic review. *Disability and Rehabilitation33*(10):797-810. DOI: 10.1016/j.pec.2010.07.046

21. Foster, G., Taylor, S.J.C., Eldridge, S., Ramsay, J., Griffiths, C.J. (2007) Self-management education programmes by lay leaders for people with chronic conditions. *Cochrane Database of Systematic Reviews*. Issue 4. [DOI: 10.1002/14651858.CD005108.pub2]

22. Hardicre, N.K. *et al.* (2018) 'An Intervention to Support Stroke Survivors and Their Carers in the Longer Term (LoTS2Care): Study Protocol for the Process

Evaluation of a Cluster Randomised Controlled Feasibility Trial', *Trials.* 19(1): 368 doi: 10.1186/s13063-018-2683-7

23. Wright, A., Burton, L., and Forster, A. (2019) *'A longer-term care strategy to support stroke survivors and their carers (LoTS2Care) – A process evaluation, the importance of contextual factors'* [online] available at: https://www.bradfordresearch.nhs.uk/wp-content/uploads/2019/03/LoTS-2-Care-Process-Evaluation.pdf

24. Cadilhac, D. A. et al. (2011) 'A phase II multicentered, single-blind, randomized, controlled trial of the stroke self-management program', Stroke; a journal of cerebral circulation, 42(6), pp. 1673–1679. DOI: 10.1016/j.pec.2010.07.046

25. McKenna, S. et al. (2015) 'Bridges self-management program for people with stroke in the community: A feasibility randomized controlled trial', International journal of stroke: official journal of the International Stroke Society, 10(5), pp. 697–704. DOI: 10.1016/j.pec.2010.07.046

26. Huijbregts, M.P, *et al*. (2008) 'Implementation, process, and preliminary outcome evaluation of two community programs for persons with stroke and their care partners' *Top Stroke Rehabil*. 15(5): 503-520. doi:10.1310/tsr1505-503

27. Taylor, D. M., Stone, S. D. and Huijbregts, M. P. (2012) 'Remote participants' experiences with a group-based stroke self-management program using videoconference technology', *Rural and remote health*, 12, p. 1947.

28. Mullis, R. et al. (2019) 'Improving Primary Care After Stroke (IPCAS) trial: protocol of a randomised controlled trial to evaluate a novel model of care for stroke survivors living in the community', Brit Med J Open, 9(8), p. E030285. DOI: 10.1016/j.pec.2010.07.046

29. Johnson, V. L., Apps, L., Hadjiconstantinou, M., Carey, M. E., Kreit E., Mullis, R., Mant, J., & Davies., M (2022) The development of a self-management intervention for stroke survivors - My Life After Stroke (MLAS), *Disability and Rehabilitation,* https://doi.org/10.1080/09638288.2022.2029959

30. Johnson, V. L., Apps, L., Kreit E., Mullis, R., Mant, J., & Davies., M on behalf of the MLAS development group (2022) The feasibility of a self-management programme (My Life After Stroke) for stroke survivors Disability and Rehabilitation doi.org/10.1080/09638288.2022.2029960

31. Aquino, M. R. J., Mullis, R., Kreit, E., Johnson, V., Grant, J., Lim, L., … & Mant, J. (2020). *Improving Primary Care After Stroke (IPCAS) randomised controlled trial: protocol for a multidimensional process evaluation.* Brit Med J open, 10(7), e036879. DOI: 10.1016/j.pec.2010.07.046

32. Ory, M,G., Jordan, P.J., Bazzarre, T. (2002) 'The Behaviour Change Consortium: setting the stage for a new century of health behaviour-change research' *Health Educ Res.* 17(5):500-511. doi:10.1093/her/17.5.500

33. Burnard, P. (1991) A method of analysing interview transcripts in qualitative research. *Nurse Educ Today*11, 461–466. https://doi.org/10.1016/0260-6917(91)90009-Y

34. Terry, G., Hayfield, N., Clarke, V. & Braun, V. (2017). *Thematic analysis. In Willig, C., & Rogers, W. The SAGE Handbook of qualitative research in psychology (pp. 17-36).* London: SAGE Publications Ltd doi: 10.4135/9781526405555

35. Clarke, V., & Braun, V. (2013). *Successful Qualitative Research: A Practical Guide for Beginners.* London: Sage.

36. Braun V, Clarke V. (2014) *What can "thematic analysis" offer health and wellbeing researchers?* Int J Qual Stud Health Well-being. 16;9:26152. doi: 10.3402/qhw.v9.26152. PMID: 25326092; PMCID: PMC4201665.

37. Forster, A. et al. (2018) 'An intervention to support stroke survivors and their carers in the longer term (LoTS2Care): study protocol for a cluster randomised controlled feasibility trial', Trials. BioMed Central, 19(1), pp. 1–14. DOI: 10.1186/s13063-018-2669-5

38. Stafford, M., et al. (2018) *"Briefing: Understanding the health care needs of people with multiple health conditions"* The Health Foundation, London.

39. Aquino, M. R. J. V., Turner, G. M., & Mant, J. (2019). Does characterising patterns of multimorbidity in stroke matter for developing collaborative care approaches in primary care?. *Primary Health Care Research & Development*, 20. doi:10.1017/S1463423619000240

40. Kim, J. I., Lee, S., & Kim, J. H. (2013). Effects of a web-based stroke education program on recurrence prevention behaviours among stroke patients: a pilot study. *Health education research*, *28*(3), 488-501. DOI: 10.1093/her/cyt044

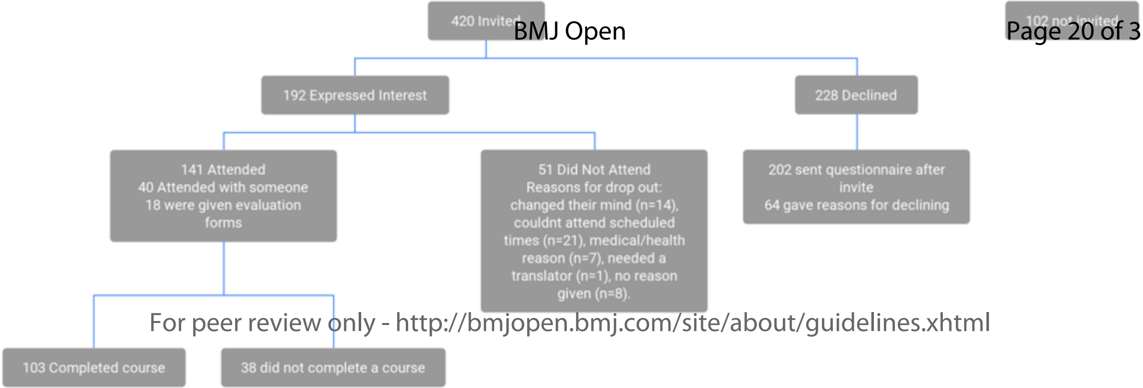

For peer review only - http://bmjopen.bmj.com/site/about/guidelines.xhtml

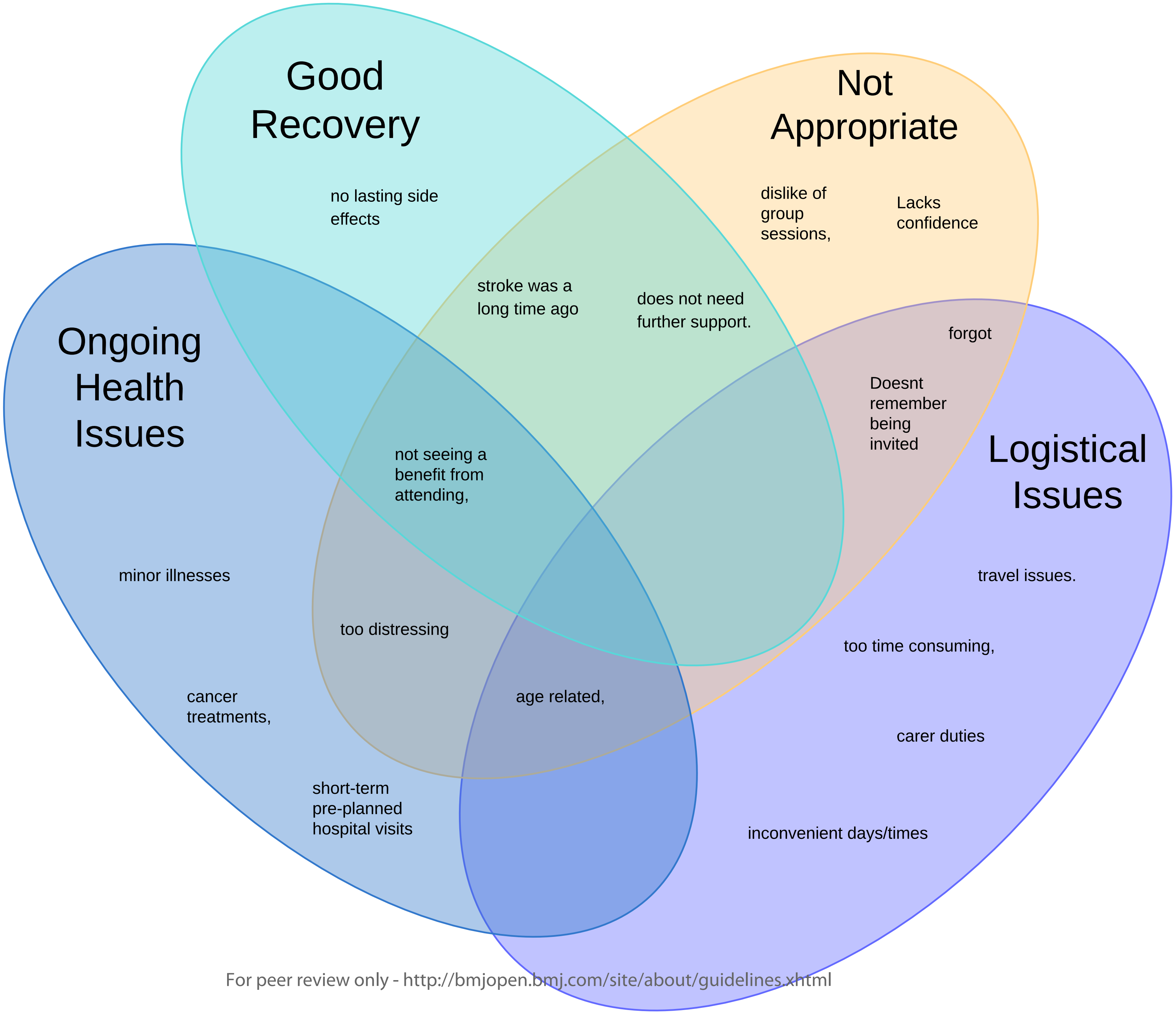
Good Recovery
Not Appropriate
Ongoing Health Issues
Logistical Issues
no lasting side effects
dislike of group sessions,
Lacks confidence
stroke was a long time ago
does not need further support.
forgot
Doesnt remember being invited
not seeing a benefit from attending,
minor illnesses
travel issues.
too distressing
too time consuming,
cancer treatments,
age related,
carer duties
short-term pre-planned hospital visits
inconvenient days/times

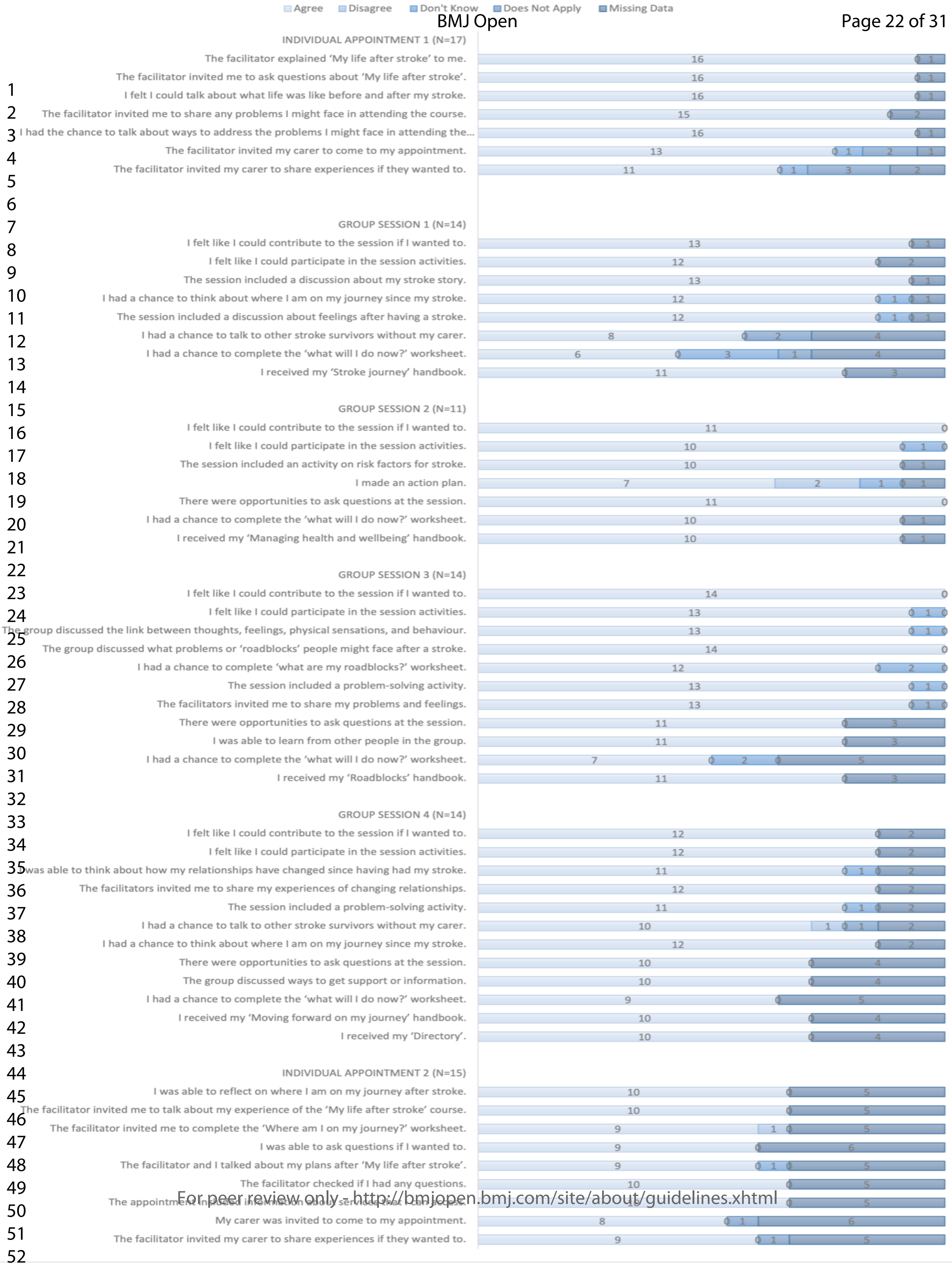

Agree
Disagree
Don't Know
Does Not Apply
Missing Data
INDIVIDUAL APPOINTMENT 1 (N=17)
The facilitator explained 'My life after stroke' to me.
The facilitator invited me to ask questions about 'My life after stroke'.
I felt I could talk about what life was like before and after my stroke.
The facilitator invited me to share any problems I might face in attending the course.
I had the chance to talk about ways to address the problems I might face in attending the...
The facilitator invited my carer to come to my appointment.
The facilitator invited my carer to share experiences if they wanted to.
GROUP SESSION 1 (N=14)
I felt like I could contribute to the session if I wanted to.
I felt like I could participate in the session activities.
The session included a discussion about my stroke story.
I had a chance to think about where I am on my journey since my stroke.
The session included a discussion about feelings after having a stroke.
I had a chance to talk to other stroke survivors without my carer.
I had a chance to complete the 'what will I do now?' worksheet.
I received my 'Stroke journey' handbook.
GROUP SESSION 2 (N=11)
I felt like I could contribute to the session if I wanted to.
I felt like I could participate in the session activities.
The session included an activity on risk factors for stroke.
I made an action plan.
There were opportunities to ask questions at the session.
I had a chance to complete the 'what will I do now?' worksheet.
I received my 'Managing health and wellbeing' handbook.
GROUP SESSION 3 (N=14)
I felt like I could contribute to the session if I wanted to.
I felt like I could participate in the session activities.
The group discussed the link between thoughts, feelings, physical sensations, and behaviour.
The group discussed what problems or 'roadblocks' people might face after a stroke.
I had a chance to complete 'what are my roadblocks?' worksheet.
The session included a problem-solving activity.
The facilitators invited me to share my problems and feelings.
There were opportunities to ask questions at the session.
I was able to learn from other people in the group.
I had a chance to complete the 'what will I do now?' worksheet.
I received my 'Roadblocks' handbook.
GROUP SESSION 4 (N=14)
I felt like I could contribute to the session if I wanted to.
I felt like I could participate in the session activities.
I was able to think about how my relationships have changed since having had my stroke.
The facilitators invited me to share my experiences of changing relationships.
The session included a problem-solving activity.
I had a chance to talk to other stroke survivors without my carer.
I had a chance to think about where I am on my journey since my stroke.
There were opportunities to ask questions at the session.
The group discussed ways to get support or information.
I had a chance to complete the 'what will I do now?' worksheet.
I received my 'Moving forward on my journey' handbook.
I received my 'Directory'.
INDIVIDUAL APPOINTMENT 2 (N=15)
I was able to reflect on where I am on my journey after stroke.
The facilitator invited me to talk about my experience of the 'My life after stroke' course.
The facilitator invited me to complete the 'Where am I on my journey?' worksheet.
I was able to ask questions if I wanted to.
The facilitator and I talked about my plans after 'My life after stroke'.
The facilitator checked if I had any questions.
My carer was invited to come to my appointment.
The facilitator invited my carer to share experiences if they wanted to.

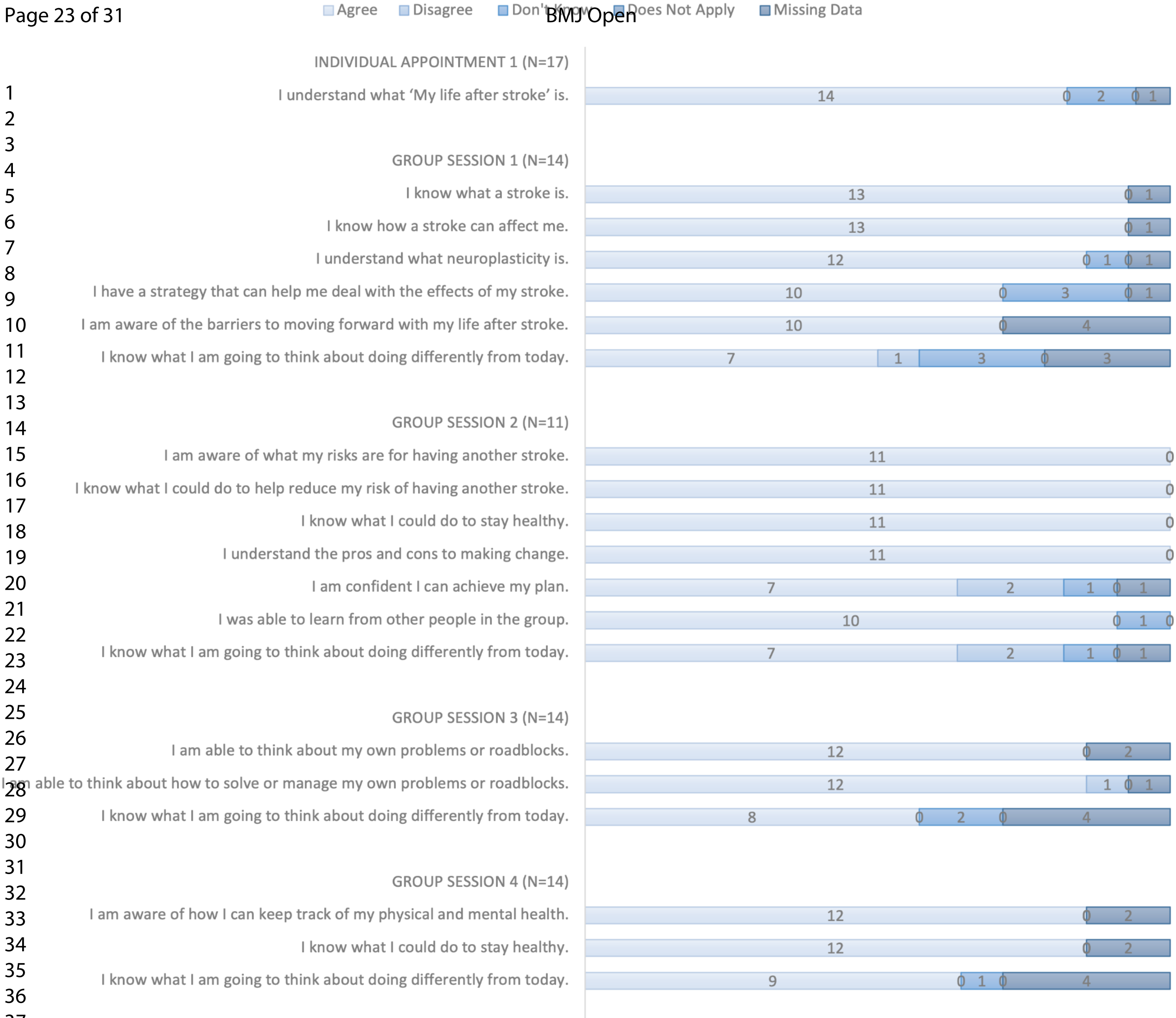
Agree
Disagree
Don't Know
Does Not Apply
Missing Data
INDIVIDUAL APPOINTMENT 1 (N=17)
I understand what 'My life after stroke' is.
14 0 2 0 1
GROUP SESSION 1 (N=14)
I know what a stroke is.
13 0 1
I know how a stroke can affect me.
13 0 1
I understand what neuroplasticity is.
12 0 1 0 1
I have a strategy that can help me deal with the effects of my stroke.
10 0 3 0 1
I am aware of the barriers to moving forward with my life after stroke.
10 0 4
I know what I am going to think about doing differently from today.
7 1 3 0 3
GROUP SESSION 2 (N=11)
I am aware of what my risks are for having another stroke.
11 0
I know what I could do to help reduce my risk of having another stroke.
11 0
I know what I could do to stay healthy.
11 0
I understand the pros and cons to making change.
11 0
I am confident I can achieve my plan.
7 2 1 0 1
I was able to learn from other people in the group.
10 0 1 0
I know what I am going to think about doing differently from today.
7 2 1 0 1
GROUP SESSION 3 (N=14)
I am able to think about my own problems or roadblocks.
12 0 2
I am able to think about how to solve or manage my own problems or roadblocks.
12 1 0 1
I know what I am going to think about doing differently from today.
8 0 2 0 4
GROUP SESSION 4 (N=14)
I am aware of how I can keep track of my physical and mental health.
12 0 2
I know what I could do to stay healthy.
12 0 2
I know what I am going to think about doing differently from today.
9 0 1 0 4

As part of the IPCAS trial you were invited by your GP or Practice Nurse to take part in the **My Life After Stroke (MLAS)** programme.

If you decided **not** to take part, please can you tell us why?

____

____

____

____

____

For peer review only - http://bmjopen.bmj.com/site/about/guidelines.xhtml

Thank you for attending **Stroke Journey**.

Please tick (✓) **agree, disagree, don't know** or **does not apply** for each of the statements below.

	Agree	Disagree	Don't know	Does not apply
1. I felt like I could contribute to the session if I wanted to.				
2. I felt like I could participate in the session activities.				
3. The session included a discussion about my **stroke story**.				
4. I had a chance to think about where I am on my **journey** since my stroke.				
5. I know what a stroke is.				
6. I know how a stroke can affect me.				
7. I understand what **neuroplasticity** is.				
8. I have a **strategy** that can help me deal with the effects of my stroke.				
9. The session included a discussion about **feelings** after having a stroke.				

Please turn over.

Improving primary care after stroke (IPCAS): A cluster randomised controlled trial
MLAS Patient Receipt Checklist – Group 1
IRAS Number: 233891 v.1.0 21-06-18

Please tick ✓ **agree**, **disagree**, **don't know** or **does not apply** for each of the statements below.

	Agree	Disagree	Don't know	Does not apply
10. I am aware of the **barriers** to moving forward with my life after stroke.				
11. I had a chance to talk to other stroke survivors **without my carer**.				
12. I had a chance to complete the **'what will I do now?'** worksheet.				
13. I know what I am going to think about doing differently from today.				
14. I received my **'Stroke journey'** handbook.				

Please write your **comments** below:

Improving primary care after stroke (IPCAS): A cluster randomised controlled trial
MLAS Patient Receipt Checklist – Group 1
IRAS Number: 233891 v.1.0 21-06-18

Thank you for attending **Managing health and wellbeing**.

Please tick (✓) **agree, disagree, don't know** or **does not apply** for each of the statements below.

	Agree	Disagree	Don't know	Does not apply
1. I felt like I could contribute to the session if I wanted to.				
2. I felt like I could participate in the session activities.				
3. The session included an activity on **risk factors for stroke**.				
4. I am aware of what my risks are for having another stroke.				
5. I know what I could do to help **reduce** my risk of having another stroke.				
6. I know what I could do to **stay healthy**.				
7. I understand the **pros and cons** to making change.				
8. I made an **action plan**.				
9. I am confident I can achieve my plan.				

Please turn over.

Please tick ✓ **agree**, **disagree**, **don't know** or **does not apply** for each of the statements below.

	Agree	Disagree	Don't know	Does not apply
10. There were opportunities to **ask questions** at the session.				
11. I was able to learn from other people in the group.				
12. I had a chance to complete the '**what will I do now?'** worksheet.				
13. I know what I am going to think about doing differently from today.				
14. I received my **'Managing health and wellbeing'** handbook.				

Please write your **comments** below:

For peer review only - http://bmjopen.bmj.com/site/about/guidelines.xhtml

Thank you for attending **Roadblocks**.

Please tick (✓) **agree, disagree, don't know** or **does not apply** for each of the statements below.

	Agree	Disagree	Don't know	Does not apply
1. I felt like I could contribute to the session if I wanted to.				
2. I felt like I could participate in the session activities.				
3. The group discussed the link between **thoughts, feelings, physical sensations, and behaviour**.				
4. The group discussed what problems or **'roadblocks'** people might face after a stroke.				
5. I had a chance to complete **'what are my roadblocks?'** worksheet.				
6. The session included a **problem-solving** activity.				
7. The facilitators invited me to share my problems and feelings.				
8. I am able to think about my own problems or roadblocks.				
9. I am able to think about how to solve or manage my own problems or roadblocks.				

Please turn over.

Improving primary care after stroke (IPCAS): A cluster randomised controlled trial
MLAS Patient Receipt Checklist – Group 3
IRAS Number: 233891
v.1.0 21-06-18

Please tick ✓ **agree**, **disagree**, **don't know** or **does not apply** for each of the statements below.

	Agree	Disagree	Don't know	Does not apply
10. There were opportunities to **ask questions** at the session.				
11. I was able to learn from other people in the group.				
12. I had a chance to complete the '**what will I do now?'** worksheet.				
13. I know what I am going to think about doing differently from today.				
14. I received my **'Roadblocks'** handbook.				

Please write your **comments** below:

Improving primary care after stroke (IPCAS): A cluster randomised controlled trial
MLAS Patient Receipt Checklist – Group 3
IRAS Number: 233891
v.1.0 21-06-18

Thank you for attending **'Moving forward on my journey'**.

Please tick (✓) **agree, disagree, don't know** or **does not apply** for each of the statements below.

	Agree	Disagree	Don't know	Does not apply
1. I felt like I could contribute to the session if I wanted to.				
2. I felt like I could participate in the session activities.				
3. I was able to think about how my relationships have changed since having had my stroke.				
4. The facilitators invited me to share my experiences of changing relationships.				
5. The session included a **problem-solving** activity.				
6. I had a chance to talk to other stroke survivors **without my carer**.				
7. I had a chance to think about where I am on my **journey** since my stroke.				
8. I am aware of how I can keep track of my **physical and mental health**.				
9. I know what I could do to **stay healthy**.				

Please turn over.

Improving primary care after stroke (IPCAS): A cluster randomised controlled trial
MLAS Patient Receipt Checklist – Group 4
IRAS Number: 233891
v.1.0 21-06-18

Please tick ✓ **agree**, **disagree**, **don't know** or **does not apply** for each of the statements below.

	Agree	Disagree	Don't know	Does not apply
10. There were opportunities to **ask questions** at the session.				
11. The group discussed ways to get **support or information**.				
12. I had a chance to complete the '**what will I do now?'** worksheet.				
13. I know what I am going to think about doing differently from today.				
14. I received my **'Moving forward on my journey'** handbook.				
15. I received my **'Directory'**.				

Please write your **comments** below:

Improving primary care after stroke (IPCAS): A cluster randomised controlled trial
MLAS Patient Receipt Checklist – Group 4
IRAS Number: 233891
v.1.0 21-06-18

Thank you for attending **Individual appointment 1**.

Please tick (✓) **agree, disagree, don't know** or **does not apply** for each of the statements below.

	Agree	Disagree	Don't know	Does not apply
1. The facilitator explained **'My life after stroke'** to me.				
2. The facilitator invited me to **ask questions** about 'My life after stroke'.				
3. I understand what **'My life after stroke'** is.				
4. I felt I could talk about what life was like **before and after** my stroke.				
5. The facilitator invited me to share any **problems I might face** in attending the course.				
6. I had the chance to talk about **ways to address** the problems I might face in attending the course.				
7. The facilitator invited my carer to come to my appointment.				
8. The facilitator invited my carer to **share experiences** if they wanted to.				

Please turn over.

Please write your **comments** below:

For peer review only - http://bmjopen.bmj.com/site/about/guidelines.xhtml

Thank you for attending **Individual appointment 2**.

Please tick (✓) **agree, disagree, don't know** or **does not apply** for each of the statements below.

	Agree	Disagree	Don't know	Does not apply
1. I was able to reflect on where I am on my **journey** after stroke.				
2. The facilitator invited me to talk about my experience of the **'My life after stroke'** course.				
3. The facilitator invited me to complete the **'Where am I on my journey?'** worksheet.				
4. I was able to **ask questions** if I wanted to.				
5. The facilitator and I talked about my **plans** after 'My life after stroke'.				
6. The facilitator checked if I had any questions.				
7. The appointment included **information about services** that I can access.				

Please turn over.

Please tick ✓ **agree**, **disagree**, **don't know** or **does not apply** for each of the statements below.

	Agree	Disagree	Don't know	Does not apply
8. My carer was invited to come to my appointment.				
9. The facilitator invited my carer to **share experiences** if they wanted to.				

Please write your **comments** below:

CONSORT 2010 checklist of information to include when reporting a randomised trial*

Section/Topic	Item No	Checklist item	Reported on page No
**Title and abstract**			
	1a	Identification as a randomised trial in the title	1
	1b	Structured summary of trial design, methods, results, and conclusions (for specific guidance see CONSORT for abstracts)	2
**Introduction**			
Background and objectives	2a	Scientific background and explanation of rationale	4
	2b	Specific objectives or hypotheses	4
**Methods**			
Trial design	3a	Description of trial design (such as parallel, factorial) including allocation ratio	5
	3b	Important changes to methods after trial commencement (such as eligibility criteria), with reasons	5
Participants	4a	Eligibility criteria for participants	5
	4b	Settings and locations where the data were collected	5
Interventions	5	The interventions for each group with sufficient details to allow replication, including how and when they were actually administered	5
Outcomes	6a	Completely defined pre-specified primary and secondary outcome measures, including how and when they were assessed	5,6
	6b	Any changes to trial outcomes after the trial commenced, with reasons	5,6
Sample size	7a	How sample size was determined	5,6
	7b	When applicable, explanation of any interim analyses and stopping guidelines	5,6
Randomisation:			
Sequence generation	8a	Method used to generate the random allocation sequence	5,6
	8b	Type of randomisation; details of any restriction (such as blocking and block size)	5,6
Allocation concealment mechanism	9	Mechanism used to implement the random allocation sequence (such as sequentially numbered containers), describing any steps taken to conceal the sequence until interventions were assigned	5,6
Implementation	10	Who generated the random allocation sequence, who enrolled participants, and who assigned participants to interventions	5,6
Blinding	11a	If done, who was blinded after assignment to interventions (for example, participants, care providers, those	n/a

For peer review only

For peer review only - http://bmjopen.bmj.com/site/about/guidelines.xhtml

		assessing outcomes) and how	
	11b	If relevant, description of the similarity of interventions	5,6
Statistical methods	12a	Statistical methods used to compare groups for primary and secondary outcomes	5,6
	12b	Methods for additional analyses, such as subgroup analyses and adjusted analyses	5,6
**Results**			
Participant flow (a diagram is strongly recommended)	13a	For each group, the numbers of participants who were randomly assigned, received intended treatment, and were analysed for the primary outcome	7
	13b	For each group, losses and exclusions after randomisation, together with reasons	7
Recruitment	14a	Dates defining the periods of recruitment and follow-up	7
	14b	Why the trial ended or was stopped	7
Baseline data	15	A table showing baseline demographic and clinical characteristics for each group	7
Numbers analysed	16	For each group, number of participants (denominator) included in each analysis and whether the analysis was by original assigned groups	7
Outcomes and estimation	17a	For each primary and secondary outcome, results for each group, and the estimated effect size and its precision (such as 95% confidence interval)	7
	17b	For binary outcomes, presentation of both absolute and relative effect sizes is recommended	7
Ancillary analyses	18	Results of any other analyses performed, including subgroup analyses and adjusted analyses, distinguishing pre-specified from exploratory	7,8,9,10,11,12,13
Harms	19	All important harms or unintended effects in each group (for specific guidance see CONSORT for harms)	
**Discussion**			
Limitations	20	Trial limitations, addressing sources of potential bias, imprecision, and, if relevant, multiplicity of analyses	13
Generalisability	21	Generalisability (external validity, applicability) of the trial findings	13
Interpretation	22	Interpretation consistent with results, balancing benefits and harms, and considering other relevant evidence	13
**Other information**			
Registration	23	Registration number and name of trial registry	16
Protocol	24	Where the full trial protocol can be accessed, if available	16
Funding	25	Sources of funding and other support (such as supply of drugs), role of funders	16

*We strongly recommend reading this statement in conjunction with the CONSORT 2010 Explanation and Elaboration for important clarifications on all the items. If relevant, we also recommend reading CONSORT extensions for cluster randomised trials, non-inferiority and equivalence trials, non-pharmacological treatments, herbal interventions, and pragmatic trials. Additional extensions are forthcoming: for those and for up to date references relevant to this checklist, see www.consort-statement.org.

For peer review only - http://bmjopen.bmj.com/site/about/guidelines.xhtml

BMJ Open

Patient experience of and participation in a stroke self-management programme, My Life After Stroke (MLAS): a multi-method study

Journal:	*BMJ Open*
Manuscript ID	bmjopen-2022-062700.R1
Article Type:	Original research
Date Submitted by the Author:	15-Aug-2022
Complete List of Authors:	Blatchford, Emily; University of Cambridge Primary Care Unit, Primary Care Unit<br>Aquino, Maria; NIHR Applied Research Collaboration North East and North Cumbria, Population Health Sciences Institute<br>Grant, Julie; University of Cambridge Primary Care Unit, Primary Care Unit<br>Johnson, Vicki; Univ Hosp Leicester NHS Trust, Leicester Diabetees Centre<br>Mullis, Ricky; University of Cambridge Primary Care Unit, Primary Care Unit<br>Lim, Lisa; University of Cambridge Primary Care Unit, Primary Care Unit<br>Mant, Jonathan; University of Cambridge Primary Care Unit, General Practice and Primary Care Research Unit
**Primary Subject Heading**:	Public health
Secondary Subject Heading:	Public health, Research methods
Keywords:	Stroke < NEUROLOGY, PRIMARY CARE, PUBLIC HEALTH

SCHOLARONE™
Manuscripts

I, the Submitting Author has the right to grant and does grant on behalf of all authors of the Work (as defined in the below author licence), an exclusive licence and/or a non-exclusive licence for contributions from authors who are: i) UK Crown employees; ii) where BMJ has agreed a CC-BY licence shall apply, and/or iii) in accordance with the terms applicable for US Federal Government officers or employees acting as part of their official duties; on a worldwide, perpetual, irrevocable, royalty-free basis to BMJ Publishing Group Ltd ("BMJ") its licensees and where the relevant Journal is co-owned by BMJ to the co-owners of the Journal, to publish the Work in this journal and any other BMJ products and to exploit all rights, as set out in our licence.

The Submitting Author accepts and understands that any supply made under these terms is made by BMJ to the Submitting Author unless you are acting as an employee on behalf of your employer or a postgraduate student of an affiliated institution which is paying any applicable article publishing charge ("APC") for Open Access articles. Where the Submitting Author wishes to make the Work available on an Open Access basis (and intends to pay the relevant APC), the terms of reuse of such Open Access shall be governed by a Creative Commons licence – details of these licences and which Creative Commons licence will apply to this Work are set out in our licence referred to above.

Other than as permitted in any relevant BMJ Author's Self Archiving Policies, I confirm this Work has not been accepted for publication elsewhere, is not being considered for publication elsewhere and does not duplicate material already published. I confirm all authors consent to publication of this Work and authorise the granting of this licence.

For peer review only

Patient experience of and participation in a stroke self-management programme, My Life After Stroke (MLAS): a multi-method study

Authors:

Emily Grace Blatchford, MA, BSc[1]
Maria Raisa Jessica (Ryc) Aquino, PhD [1,2]
Julie Grant, BSc [1]
Vicki Johnson, BSc [3]
Ricky Mullis, PhD[1]
Lisa Lim, MB BChir[1]
Jonathan Mant, MD[1]

1 Primary Care Unit, Department of Public Health and Primary Care, University of Cambridge, UK

2 NIHR Applied Research Collaboration North East & North Cumbria, Population Health Sciences Institute, Faculty of Medical Sciences, Newcastle University, UK

3 Leicester Diabetes Centre, University Hospitals Leicester NHS Trust, UK

Corresponding author:

Emily Grace Blatchford
egb39@cam.ac.uk
Primary Care Unit, Department of Public Health and Primary Care, University of Cambridge, UK

For peer review only

Abstract

Objective
A self-management programme, My Life After Stroke (MLAS), was developed to support stroke survivors. This evaluation reports patient experience.

Design
Multi method, involving interviews and questionnaires

Setting
23 general practices in the intervention arm of a cluster randomised controlled trial in East of England and East Midlands, UK

Participants
People on the stroke registers of participating general practices were invited to attend an MLAS programme.

Interventions
MLAS comprises one-to-one and group-based sessions to promote independence, confidence, and hope.

Primary and secondary outcome measures
The primary outcome was uptake of the programme. Participants who declined MLAS were sent a questionnaire to ascertain why. Attendees of four programmes completed evaluation forms. Attendees and Non-attendees of MLAS were interviewed. Ad-hoc email conversations with the lead author were reviewed. Thematic analysis was used for qualitative data.

Results
141/420 (34%) of participants (mean age 71) attended an MLAS programme and 103 (73%) completed one. 64/228 (28%) participants who declined MLAS gave reasons as: good recovery; ongoing health issues; logistical issues; inappropriate. Nearly all attendees who completed questionnaires felt that process criteria such as talking about their stroke and outcomes such as developing a strong understanding of stroke had been achieved.

Conclusions
MLAS was a positive experience for participants but many stroke survivors did not feel it was appropriate for them. Participation in self-management programmes after stroke might be improved by offering them sooner after the stroke and providing a range of delivery options beyond group-based, face-to-face learning.

For peer review only

Article Summary

Strengths and Limitations

- The self-management programme used in this study was developed using extensive patient and public involvement, and has been demonstrated to be feasible to deliver
- The study population is people on general practice stroke registers rather than those recruited from specialist stroke services, so better reflects the experience of a self-management programme of longer-term community dwelling stroke survivors
- The study does not provide data on effectiveness of this self-management programme, but on uptake and patient experience
- The multi-methods approach allowed us to understand the reasons that people do not take part in these programmes.

For peer review only

Introduction

Background

There is a lack of support for stroke survivors living in the community[1,2,3]. Stroke survivors report a sense of abandonment and a need for social, psychological, and educational support[4,5,6,7]. Psychological issues are associated with higher rates of mortality, long-term disability, hospital readmissions, suicide and higher utilisation of outpatient services[8,9,10]. The need for development and evaluation of different models of care to address long-term needs of stroke survivors in primary care is well recognised[11], yet such models remain scarce[12]. Adjusting to life after stroke continues after formal rehabilitation has finished[13,14]. The evidence-base for the role of self-management programmes is growing[15]: such approaches have been successfully applied to long-term conditions such as diabetes[16,17] and can reduce pressure on social and healthcare services[18] by allowing participants to take an active role in their own condition, making informed choices whilst adopting behaviour and psychological changes[15,19,20,21].

Self-management programmes have been used to support stroke survivors during their aftercare. Programmes such as the New Start[22,23] intervention, the Australia-based Stroke Self-Management Programme (SSMP)[24], Bridges[25], and the Canadian Moving On after STroke (MOST)[26] include processes such as increasing contact time, identifying needs, exploring social networking, problem-solving, goal setting, forming action plans, providing stroke-specific information, using trained healthcare professionals and increasing exercise. MOST-Telehealth Remote[27] took self-management virtually using video conferencing to remotely connect facilitators with participants further afield.

As part of a new primary care-based model of care, Improving Primary Care After Stroke (IPCAS)[28], a novel self-management programme, My Life After Stroke (MLAS) was developed using the MRC framework and extensive patient and public involvement (PPI), to target the long-term needs of stroke survivors[29]. Areas covered include social wellbeing, community re-integration, emotional support, physical needs, activities of daily living and prevention of future strokes, informed by self-efficacy and cognitive behavioural therapy (CBT). This intervention is one of few that focus on stroke survivor self-reported needs. MLAS was subsequently found to be feasible to deliver[30], and so was included as one of the components of IPCAS that was evaluated in a randomised controlled trial[28].

Objective

While it was not possible to directly evaluate any of the individual components within the IPCAS model of care using the randomised design, we wanted to better understand the potential role of self-management programmes such as MLAS. This paper therefore focuses on patient experience of and participation in MLAS as part of a larger process evaluation[31].

Methods

MLAS

MLAS programmes (n=24) were held between September 2018 and January 2020 across six counties in England: Norfolk (n=8), Suffolk (n=4), Northamptonshire (n=4), Cambridgeshire (n=5), Leicestershire (n=2) and Essex (n=1). Programmes lasted nine weeks with over eleven hours' contact time[29]. There is an individual appointment with a facilitator at the start of the programme, and a second one at the end. In between individual appointments, there are four group sessions (2.5 hours each) covering different topics. The completion criterion states participants must attend both individual appointments, group session-1 and a minimum of two other group sessions. Individual appointments (face-to-face or telephone) consist of a discussion to build rapport and explore the impact of their stroke before and after MLAS. Group sessions were interactive, consisted of social support and signposting to local services and topics including managing physical and emotional health, warning signs, and risks of stroke. All sessions aimed to build confidence, independence and hope through problem-solving, Cognitive Behavioural Therapy (CBT)-based approaches, and opportunities to share personal experiences. The same two facilitators ran all six sessions of a programme (where possible) and were trained and mentored in behaviours such as being non-judgemental, not giving specific advice, effective communication and active listening skills, and how to use the MLAS facilitator curriculum and resources to deliver the intervention as intended. Participants were provided with refreshments, programme handbooks and each session began with a recap of previous sessions.

Recruitment

Participants were in the intervention arm of the IPCAS cluster randomised trial[28]. People were eligible to take part in IPCAS if they were on the stroke register of a participating general practice, were 18 years or older and able to provide written informed consent, with or without the help of a carer. They were invited to attend a programme whilst at a stroke review led by a health care professional at their GP surgery. Invitees were given the study team's contact details and the lead author (EB) contacted all participants two weeks post-invitation by telephone or email. Those expressing interest were booked onto a programme in an accessible (e.g., parking, wheelchair-friendly and accessible facilities) community venue near their general practice surgery. Pre-arranged and funded return-transport was available. Carers and/or significant others were encouraged to attend with participants.

Facilitators

Facilitators (n=23) were trained by the intervention developers during 2018/19[29]. Facilitators' backgrounds included nursing, psychology, and those with specific interest in stroke.

Methods of data collection

Qualitative and quantitative data were collected in order to answer the questions below. The qualitative data included a sub-set of interviews that had been carried out

For peer review only

for the wider process evaluation[31]. This included 8 participants who completed MLAS, 4 who started, but did not complete, and 5 who did not take part (2 of whom had expressed interest).

Why did participants decline MLAS?

In addition to interviews with 5 non-participants, a questionnaire was sent 6-months post-randomisation to all participants which included a question asking if they hadn't attended MLAS, why that was (see appendix A). In some areas, there were delays with set-up of MLAS which meant that participants' received the questionnaire before their invitation to attend the programme and so were unable to provide a response to this question.

What did participants experience when attending MLAS?

All attendees from a purposefully selected sample of programmes(n=4) received an evaluation form to complete at the end of each MLAS session which explored what happened during the session and what they took away from it. Programme selection criteria included facilitators leading programmes and geographical location. We also aimed to include participants that were not participating in other elements of the wider process evaluation (e.g., interviews) to offer individuals opportunities to share their experiences, and at the same time, minimise participant burden. Responses were on a Likert scale and included a comments box (see appendix B).

What are participant views on MLAS?

Participants in the process evaluation were invited to take part in semi-structured telephone interviews. Potential participants were purposively selected to ensure that people who completed a programme, started a programme but did not complete it, declined to take part, or agreed to take part but did not attend were all included. In order to facilitate engagement of people with aphasia or cognitive impairment, participants could select how they were interviewed (with or without carer; by telephone or face to face or by email). Interviews were performed by JG using a bespoke topic guide to aid open-ended discussion suitable for differing levels of cognitive function. The topic guide was piloted with a stroke survivor to ensure suitability of questions. Topics covered experience and participation (see appendix C), informed by the Behaviour Change Consortium framework[32].

What feedback was given after MLAS was delivered?

Informal general feedback was given to the lead author (EB) from both participants and facilitators via email post-attendance.

Analysis

We applied the six-phase approach of Braun and Clarke[33] to the data as follows: 1) data familiarisation where transcripts and questionnaires with qualitative data were read and reread; 2) generation of initial codes, where two coders independently generated codes on a subset of transcripts, then refined these and applied the codes to the rest of the data; 3) identified themes, i.e. searching for patterns within the coded data; 4) reviewing the themes to refine each theme's meaning, and to

For peer review only

determine whether some themes lacked coherence/depth and needed discarding, 5) defining and naming themes which involved finalising the set of themes, discussing any disagreements between coders with a third member of the research team (RA); and finally 6) writing up the report which was led by the first author (EB) Patient receipt questionnaires were analysed for process and outcome data and simple descriptive statistics were used for the quantitative data. A two sample *t* statistic was used to compare if there was a difference between those who attended and did not attend, in relation to the distance from individuals' home to the programme venue and in relation to time since stroke.

Patient and Public Involvement

We actively engaged PPI in the development of the Managing Life After Stroke self-management programme through lay membership of our Programme Steering Committee and our Intervention Development Group, a lay representative co-applicant, active engagement with The Stroke Association, and in several meetings with stroke survivor and carer support groups in the community.

Consultation meetings took place with stroke survivors in April 2016 (2 in Leicester, 1 in Cambridge - 20 people per group) to seek input on content of the self-management intervention and its format.

The topic guides for our interviews were pilot tested with PPI members via email, telephone calls and a mock interview.

Results

522 stroke survivors were randomised to the intervention arm. Fifty-nine did not attend the stroke review, 42 withdrew from the trial and 2 died. As a result, 420 individuals were invited to attend an MLAS programme. 192 (46%) participants who were invited to MLAS expressed interest in attending (Figure 1). Of those expressing interest, 141 (73%) attended, and 103 of those attending (73%) completed the programme. Twenty four separate MLAS programmes were held. The median attendance at a group session was 4.5 (range 2-8).

Participant characteristics are shown in Table 1. 94% (133/141) of participants attended a programme more than a year after their stroke. There were no differences between those who attended and did not attend. 70% (99/141) of participants attended a programme within 2-miles from their residence. The mean distance from the programme venue to the stroke survivor's home and time since stroke were not significantly different between those who did and did not attend the programme (*p-value* 0.26 and 0.50 respectively).

Table 1
Characteristics of stroke survivors by whether or not they attended the MLAS programme *SD = Standard deviation*

	**Attended (n=141)**	**Expressed Interest but Did Not Attend (n=51)**	**Did not wish to attend (n=228)**
**Gender**			
Male	62.4% (n=88)	62.7% (n=32)	66.7% (n=152)
Female	37.6% (n=53)	37.3% (n=19)	33.3% (n=76)

For peer review only

**Ethnicity**			
White	97.9% (n=138)	100.0% (n=51)	96.5% (n=220)
Other	2.1% (n=3)	0.0% (n=0)	3.5% (n=8)
**Age**			
Mean yearsat baseline, (SD)	71.2 (10.4)	70.9 (12.3)	69.7 (11.7)
**Time Since Stroke (tss)**			
Mean years since stroke to study entry (SD)	7.7 (9.0)	6.6 (7.3)	8.2 (8.5)
**Distance to MLAS Venue**			
Mean miles from residence to MLAS venue* (SD)	2.3 (3.6)	3.5 (5.1)	2.8 (4.0)

*Excludes patients from one practice where 13 patients were invited, but the programme was never run.

Why did participants decline MLAS?

Sixty-four participants gave multiple reasons for declining MLAS which were grouped into four main themes: Good recovery, Ongoing health issues, Logistical issues, and Not appropriate (Figure 2).

Good Recovery

28 people felt they made a good or full recovery, experienced no lasting side effects, and their stroke was a long time ago; therefore no longer needed further support.

Ongoing Health Issues

13 people had ongoing health or medical issues such as age-related concerns, cancer treatments, short-term pre-planned hospital visits or minor illnesses.

Logistical issues

9 people were unable to attend due to other commitments such as carer duties, the programme being too time consuming, inconvenient days/times and travel issues.

Not Appropriate

22 people did not feel the programme was appropriate for them, including disliking group sessions, lacking confidence, uncertainty about the benefits of attending, and concerns about the programme being too distressing.

The most common reason given by people who had indicated they would like to attend but subsequently did not was that it was being held at an inconvenient time. Views expressed on self-management programmes in general by non-attenders were positive; they thought they would be useful, tailored to individual needs, good for social aspects, and learning.

The Interviews with participants did not attend MLAS gave reasons matching those in Figure 2, with additional ones including: the programme having no connection to their stroke, previously attending similar programmes, being in full-time employment, and moving away.

For peer review only

What did participants experience when attending MLAS?

Figures 3 and 4 show the extent to which participants agreed that the programmes were carried out as intended. 18 participants were invited to complete evaluation forms. There were 54 process criteria which included learning about MLAS, talking about one's stroke, asking questions, and forming discussions. All participants who responded felt these were met with the exception of three criteria, where in each case only 1 or 2 participants felt otherwise. Similarly, there were 20 outcome criteria which were largely concerned with different aspects of knowledge about stroke. There were only two criteria which a responder felt were not met relating to confidence in building action plans and acting upon them. Free text collected from these forms (n=14) was positive and showed participants had "interesting discussions", "thoroughly enjoyed the course", found it "useful" and enjoyed "meeting fellow members and hearing their experiences". One participant said "I have really pushed myself to get past my roadblock and have started to use my quadstick to move around short distances (with somebody close by). I am sure I would not have attempted this without attending these little meetings".

What are participants' views on MLAS?

The Interviews with participants who attended MLAS generated four themes : Programme characteristics influence programme participation, programme content is relevant, perceived benefits of attendance, and personal circumstances affect programme participation.

Programme Characteristics Influence Programme Participation

Participants (n=4) spoke about the logistics of programmes, referring to preferred days of the week, time of day, location (closer to home the better) and transport (taxis benefited participants with mobility issues). Participants (n=5) also discussed the structure of group sessions (some enjoyed groups and others did not), size of groups (small groups preferred – as delivered) and length of sessions (2-hours was long enough). One participant said "I think six [participants] was quite nice" [female, 68, completed MLAS]. Another participant said: "I can't cope when there's lots of people in the room and too much information is coming in…too many people's voices" [female, 56, completed MLAS].

Programme Content Is Relevant

Despite pre-conceptions that the course content might not be relevant, participants, on engaging with the programme content, reported that it was. Some participants were sceptical about the activities, such as using a roadmap metaphor (i.e where participants were tasked with placing themselves on a printout of a road going in various directions) to aid thinking through barriers to recovery. One reported saying to their niece: "what on earth are they on about being in a bleeding taxi?" But then she went on to say: "I didn't know what my road-blocks were until I worked through the book, and the same answer kept coming up, and it was fear, fear, fear….So once I'd said in the group, my biggest thing was fear.. that was a major unblocking". Another participant: "Kept saying to myself: how relevant is that now?" But then said: "It kicked my backside! I wasn't actually doing some of the things I should be doing, like ..the clarinet..(and an) exercise bike" (male 90; completed MLAS). Participants (n=6) reported that programme content and delivery should meet attendees'

For peer review only

individual needs. One participant commented, “not everyone’s difficulties are so obvious” [female 81; partially completed MLAS] as participants had their stroke at different times in their lives resulting in various after-effects. Another participant explained, “I struggle a lot with my memory...I can forget, you know, talking to you, I’d forget your name at the end” [female 58; partially completed MLAS]. The contributions of individual participants (e.g. identifying their own road-blocks, as above) enabled content to match need. By remaining flexible in delivery, participants were able to complete handbooks at their own pace without feeling pressured: "So when it was time to do your workbooks, I said I’ll do this later, so that was never a problem. And I think I gained more from doing it after” [female, 56, completed MLAS].

Perceived Benefits of Attendance

Participants (n=6) reported a change in self-awareness, with a less negative mind-set. They also (n=4) perceived change in their behaviour, including increasing exercise and engaging in hobbies; one participant said, “I was sitting there thinking there isn’t anything I can do, but then I remembered I’d got this big purple exercise ball. So, it actually made me get it out, and I’ve got a new sewing machine...so now that’s made me buzz, because now I’m back into patchwork and quilting so I think right, I’ll join a patchwork and quilting group. So that was a very positive thing that came out of it… . I’m altogether just so much different…Creatively, I’m just buzzing” [female, 56, completed MLAS]. Thirdly, participants (n=10) reported that the programme increased their understanding of stroke and how it affected people: “People shared their experiences of it, actual strokes, what they could remember, and that to me was very interesting, realising that everybody really had had a different experience, but there were things in common that we were all grappling with” [female, 73 , completed MLAS]. Finally, participants (n=11) considered attendance to be a positive experience, gaining social support and engaging in altruism; a participant said “I found it so helpful just listening to other people’s stories and feeling able to talk about my own feelings so that was very, very helpful” [female, 78].

Personal Circumstances Affect Programme Participation

Participants (n=7) said individual circumstances affected their participation, including having existing health comorbidities and how far into recovery they were. One participant said “with my eyes it is quite difficult to do all the things that I used to do...I can’t use my legs and now I can’t use my eyes either” [female, 56] another participant said “it’s [been] five years since I had the stroke, but most people at the group, it was a longer time since they’d had their strokes…I know this is a new project, but I would have found it helpful to have had this course much nearer to the stroke” [female, 73, completed MLAS]. On the other hand, she went on to mention that there was one lady “who’d had a stroke [recently] and I felt perhaps that was too soon for her because she was still working things through, but you know, it’s probably a personal thing”.

What feedback was given after MLAS was delivered?

Informal feedback was received from participants (n=2) and facilitators (n=10) via emails after attending an MLAS programme and grouped into three themes.

For peer review only

Participants had a positive experience

Participants expressed positive experiences and found the programme useful. One said he was “sceptical at first about joining the course and felt it would be a waste of time” but has “learnt so much and is glad he attended” [70, male]. Another mentioned it was “good to speak with other stroke survivors” and found problem solving “most useful when it came to putting it into real life” [78, female]. A facilitator said that one participant didn't realise how angry he was, but by the second group session “felt like a weight had been lifted” [Northamptonshire programme]. Facilitators recognised participants were positive when completing the programmes as they had learnt from each other and shared personal experiences.

Programme more beneficial closer to time of stroke onset

Participants' strokes were often not recent, one stated that the programme “would be more useful when you come home from hospital”. Facilitators explained how participants struggled to identify areas of difficulty as they no longer fitted the stereotypical stroke survivor.

Ongoing health issues

Facilitators expressed the view that many of participants had ongoing health comorbidities which were often more pronounced than the consequences of their stroke.

Discussion and Conclusion

Key Findings

Participants who attended MLAS had a positive experience and felt that the programmes achieved what was intended. However, over half of those invited declined to attend. Reasons included: good recovery, ongoing health issues, logistical issues, or not feeling the programme was appropriate for them.

Strengths and Limitations

More participants (n=141) took part in MLAS compared to evaluations of other self-management programmes for stroke[22,23,24,26,27,34]. Using a mixed methods approach helped to identify reasons for low participation, and explore why those who had attended MLAS felt it was a success. Furthermore, consistent themes were identified from the different sources of data. All qualitative data were double coded, with a third experienced analyst resolving disagreements. Only 32% of people who declined to take part in a programme gave an explanation as to why this was, but these data were supplemented by five interviews with those who did not attend. Only a small sample of attendees received an evaluation form (13%) from four programmes (17%), to minimise participant burden. The use of emails provided a perspective from the facilitators, which complemented that of participants. However, there was a lack of diversity within the sample, participants being primarily white, retired, and more than a year post stroke. Participants may not have been representative of the whole population of stroke survivors as they were all taking part in a randomised controlled trial.

For peer review only

Comparison with existing literature

There is some evidence from randomised controlled trials that self-management programmes may improve quality of life and self-efficacy[15]. With regard to patient experience and participation, there have been insights from before and after studies and randomised control trials (RCT). The New Start intervention[34], which has similarities to MLAS, is a self-management programme for patients within the first six months of their stroke which is being tested in an ongoing RCT[22,23]. The SSMP intervention[24] was part of an RCT that aimed to recruit participants of similar age to MLAS >3months post stroke to an eight week programme; 70% of attendees were >12months post stroke, which is similar to MLAS. The MOST intervention[26] compared a new self-management programme with 34-hours of group contact time over eight weeks to a standard education programme >3months post stroke. It involved three times as much contact time as MLAS using similar sized groups and ages. MLAS does appear to have a slightly lower uptake than these programmes but a higher completion rate. The SSMP intervention[24] had 53% (27/48) attendance compared to 34% (141/420) in MLAS, with 52% (25/48) completing >50% of sessions compared to MLAS which had 90.1% of participants complete more than 50% of sessions. This could be due to participants receiving dates in advance and having transport provided. The MOST intervention[26] had a small 60% (18/30) participant recruitment and only 22% (4/18) attended all of the sessions compared to 53.2% for MLAS. Qualitative feedback from participants of these other programmes was positive, as it was for MLAS. Reasons for non-attendance of the SSMP course (time constraints, lack of interest and inability to attend e.g., because of transportation issues) were similar to MLAS. A follow up study to MOST[26], MOST-TR[27] involved video conferencing with 36-hours of online-contact time over a nine week period with participants 3-18-months post stroke. Participants valued access to this program without the need to travel. A review[15] of the effectiveness of self-management programmes for community living adults with stroke identified 14 RCTs conducted across eight countries between 2000-2015 with 1863 participants. All programmes were community, home or out-patient based and lasted a duration of four-weeks to six-months. There was some evidence of improved quality of life (the primary outcome). Secondary outcome measures showed positive improvements in self-efficacy, self-esteem, emotional stability, anxiety and depression and mood.

Interpretation

Given that there is some evidence that self-management programmes can improve quality of life in stroke survivors,[15] it is important to understand why people do or do not attend such programmes. While people who attended the MLAS programme found it helpful, many people in our sample did not feel that the programme was suitable for them. Some people felt that it had been too long since their stroke had passed to be useful to attend, though other programmes that recruited participants earlier after their stroke also had poor uptake[24,26,27]. It is a feature of primary care that most people on stroke registers will not have had a recent stroke, and so this raises the question as to whether self-management programmes might be more useful if accessed from specialist secondary care services or offered at the point of discharge or engagement with early supported discharge (ESD). Other potential participants felt they had too many ongoing health problems to attend, highlighting the problem of providing single-condition programmes for people with multimorbidity[35,36]. For those who do wish to attend a programme, it needs to be

For peer review only

adaptable to patient needs and wishes. For example, it has been shown to be feasible to provide virtual sessions which can overcome attendance barriers[37]. Tailoring programmes might also address concerns of people who do not like group work. It is possible the decision to take part in an MLAS programme was influenced by how it was introduced to the stroke survivor by the healthcare professional. It may be that uptake could be improved by focussing on this aspect. Before widespread adoption of self-management programmes such as MLAS, further evidence is required on optimal timing, content, and mode of delivery[15].

Conclusion

The mixed-methods and multi-perspective nature of this analysis has provided insights into patient experience of and participation in a stroke self-management programme, MLAS. This intervention, which focuses on addressing patient-self-reported needs, was found to provide a positive experience for participants. Although many stroke survivors who declined participation did not feel it was appropriate for them, it might have a role as part of a menu of optional services.

Practical Implications

This study suggests that there should not be an expectation that all stroke survivors would want to take part in a self-management programme such as MLAS. There was feedback that the programme would be more valuable if offered sooner after the stroke. This could be achieved by having referring routes from specialist services rather than from primary care, though uptake is not necessarily higher from these settings. Some potential participants did not like the idea of group work, and others found it logistically difficult to attend. Setting up on-line sessions (whether individual or group based) would be a way of addressing such concerns.

Figure Legend

Figure 1 - Study flow chart for those invited, attended and declined MLAS
Figure 2 - Venn diagram of themes for declining the self-management programme, MLAS
Figure 3 - Patient evaluation forms, Process questions. n= number of participants attending the session
Figure 4 - Patient evaluation forms; Outcome questions. n= number of participants attending the session – Individual appointment 2 had no outcome questions

Acknowledgements

We are thankful for the IPCAS administration team members who included Lizzie Kreit, Victoria Theobald and Alex Newbrook, and to James Brimicombe for his database work. With thanks to the trainers from Leicester and the MLAS facilitators, who without them, the programmes would not have been delivered in the community venues across England. Thank you to the stroke survivors participating in the study. Thank you to Bundy Mackintosh, patient adviser.

Funding

This study is funded by the National Institute for Health Research's Programme Grant for Applied Research titled 'Developing primary care services for stroke survivors' reference PTC-RP-PG-0213-20001. The views expressed are those of the authors and not necessarily those of the NIHR or the Department of Health and Social Care. Jonathan Mant is an NIHR Senior Investigator.

Declaration of competing interests

Vicki Johnson declares that she is employed by the University Hospitals of Leicester NHS Trust within the Leicester Diabetes Centre (LDC), which receives not-for-profit income for DESMOND, a suite of self-management programmes for which LDC holds the Intellectual Property Rights for. UHL also receives various grants to pay staff within LDC to carry out and implement various studies and self-management programmes. The Intellectual Property for MLAS is held by University of Leicester on behalf of UHL. All other authors declare that they have no competing interests.

Author Statement

- Emily Grace Blatchford: conceptualization; methodology, investigation, data curation; writing- original draft; visualisation.
- Maria Raisa Jessica Aquino: conceptualization; methodology; writing – review & editing; project administration; supervision.
- Julie Grant: investigation; data curation; writing - review & editing.
- Vicki Johnson: conceptualization; resources; writing – review & editing.
- Ricky Mullis: conceptualization; writing – review & editing; supervision; project administration; funding acquisition.
- Lisa Lim: conceptualization; writing – review & editing
- Jonathan Mant: conceptualization; writing – review & editing; supervision; funding acquisition.

Trial Registration

IPCAS ClinicalTrials.gov identifier (NCT number): ***NCT03353519***

Data Statement

Data are available upon reasonable request. The data may be available on request from the authors, subject to appropriate permissions: please email the PI Jonathan Mant (jm677@medschl.cam.ac.uk) for details.

For peer review only

Ethics Statement

It does involve human participants and has been approved by an Ethics Committee, Yorkshire & The Humber-Bradford Leeds NHS Research Ethics Committee (17/YH/0441)

For peer review only

References

1. National Audit Office (2010). Progress in improving stroke care: Department of Health, HC 291, Report by Comptroller and Auditor General, Session 2009–10. Department of Health: Progress in improving stroke care - National Audit Office (NAO) Report

2. McKevitt, C., Fudge, N., Redfern, J., Sheldenkar, A., Crichton, S., Rudd, A. R., ... & Wolfe, C. D. (2011). Self-reported long-term needs after stroke. *Stroke*, *42*(5), 1398-1403. DOI: 10.1161/STROKEAHA.110.598839

3. Pindus, D. M., Mullis, R., Lim, L., Wellwood, I., Rundell, A. V., Abd Aziz, N. A., & Mant, J. (2018) 'Stroke survivors' and informal caregivers' experiences of primary care and community healthcare services – A systematic review and meta-ethnography', *Plos One*, 13(4). doi: 10.1371/journal.pone.0192533.

4. Hafsteinsdóttir, T. B., Vergunst, M., Lindeman, E., & Schuurmans, M. (2011) 'Educational needs of patients with a stroke and their caregivers: A systematic review of the literature', *Patient education and counselling*. Elsevier, *85*(1), pp. 14–25. DOI: 10.1016/j.pec.2010.07.046

5. Taylor, S. J. C. *et al.* (2014) 'A rapid synthesis of the evidence on interventions supporting self-management for people with long-term conditions: PRISMS – Practical systematic RevIew of Self-Management Support for long-term conditions', *Health Services and Delivery Research*, 2(53), pp. 1–580. DOI: 10.1016/j.pec.2010.07.046

6. White, C. L., Barrientos, R. and Dunn, K. (2014) 'Dimensions of Uncertainty After Stroke', *Journal of Neuroscience Nursing*, 46(4), pp. 233–240. DOI: 10.1016/j.pec.2010.07.046

7. Chun, H.-Y. Y. *et al.* (2018) 'Anxiety After Stroke: The Importance of Subtyping', *Stroke; a journal of cerebral circulation*, 49(3), pp. 556–564. DOI: 10.1016/j.pec.2010.07.046

8. Allida S, Cox KL, Hsieh C-F, House A, Hackett ML. (2020) Pharmacological, psychological and non-invasive brain stimulation interventions for preventing depression after stroke.

9. Towfighi A, Ovbiagele B, Husseini NE et al on behalf of the American Heart Association Stroke Council; Council on Cardiovascular and Stroke Nursing; and Council on Quality of Care and Outcomes Research. (2017) Post-stroke depression: a scientific statement for healthcare professionals from the American Heart Association/American Stroke Association. *Stroke* 48:e30-e43.

10. Williams, L. S. (2005) 'Depression and Stroke: Cause or Consequence?', *Seminars in Neurology*, 25(04), pp. 396–409. DOI: 10.1016/j.pec.2010.07.046

For peer review only

11. Williams, L. S. and Rudd, A. G. (2010) 'Advances in health policy and outcomes 2009', *Stroke; a journal of cerebral circulation*, 41(2), pp. E77–80. DOI: 10.1016/j.pec.2010.07.046

12. Lennon, S., McKenna, S. and Jones, F. (2013) 'Self-management programmes for people post stroke: a systematic review', *Clinical Rehabilitation*, 27(10), pp. 867–878. DOI: 10.1016/j.pec.2010.07.046

13. Cott, C. A., Wiles, R., Devitt, R. (2007) Continuity, transition and participation: preparing clients for life in the community post stroke. *Disability and Rehabilitation* 29(20-21):1566-74. DOI: 10.1016/j.pec.2010.07.046

14. Pound, P., Gompertz, P., Ebrahim, S. (1998) A patient-centred study of the consequences of stroke. *Clinical Rehabilitation* 12:255-64. DOI: 10.1016/j.pec.2010.07.046

15. Fryer, C.E., Luker, J.A., McDonnell, M.N., Hillier, S.L. (2016) Self-management programmes for quality of life in people with stroke. *Cochrane Database of Systematic Reviews*. Issue 8. Art. No.: CD010442. DOI: 10.1002/14651858.CD010442.pub2.

16. Skinner, T. C. et al. (2006) 'Diabetes education and self-management for ongoing and newly diagnosed (DESMOND): Process modelling of pilot study', Patient Education and Counselling, 64(1-3), pp. 369–377. DOI: 10.1016/j.pec.2010.07.046

17. Davies, M. J. et al. (2008) 'Effectiveness of the diabetes education and self-management for ongoing and newly diagnosed (DESMOND) programme for people with newly diagnosed type 2 diabetes: cluster randomised controlled trial', Brit Med J , 336(7642), pp. 491–495. DOI: 10.1016/j.pec.2010.07.046

18. de Silva, D. (2011) Evidence: Helping people help themselves: A review of the evidence considering whether it is worthwhile to support self-management. The Evidence Centre.

19. Lorig, K.R., Mazonson, P.D., Holman, H.R.. (1993) Evidence suggesting that health education for self-management in patients with chronic arthritis has sustained health benefits while reducing health care costs. *Arthritis & Rheumatism* 36(4):439-46. DOI: 10.1016/j.pec.2010.07.046

20. Jones, F., Riazi, A. (2011) Self-efficacy and self-management after stroke: a systematic review. *Disability and Rehabilitation33*(10):797-810. DOI: 10.1016/j.pec.2010.07.046

21. Foster, G., Taylor, S.J.C., Eldridge, S., Ramsay, J., Griffiths, C.J. (2007) Self-management education programmes by lay leaders for people with chronic conditions. *Cochrane Database of Systematic Reviews*. Issue 4. [DOI: 10.1002/14651858.CD005108.pub2]

For peer review only

22. Hardicre, N.K. *et al.* (2018) 'An Intervention to Support Stroke Survivors and Their Carers in the Longer Term (LoTS2Care): Study Protocol for the Process Evaluation of a Cluster Randomised Controlled Feasibility Trial', *Trials.* 19(1): 368 doi: 10.1186/s13063-018-2683-7

23. Wright, A., Burton, L., and Forster, A. (2019) *'A longer-term care strategy to support stroke survivors and their carers (LoTS2Care) – A process evaluation, the importance of contextual factors'* [online] available at: https://www.bradfordresearch.nhs.uk/wp-content/uploads/2019/03/LoTS-2-Care-Process-Evaluation.pdf

24. Cadilhac, D. A. et al. (2011) 'A phase II multicentered, single-blind, randomized, controlled trial of the stroke self-management program', Stroke; a journal of cerebral circulation, 42(6), pp. 1673–1679. DOI: 10.1016/j.pec.2010.07.046

25. McKenna, S. et al. (2015) 'Bridges self-management program for people with stroke in the community: A feasibility randomized controlled trial', International journal of stroke: official journal of the International Stroke Society, 10(5), pp. 697–704. DOI: 10.1016/j.pec.2010.07.046

26. Huijbregts, M.P, *et al*. (2008) 'Implementation, process, and preliminary outcome evaluation of two community programs for persons with stroke and their care partners' *Top Stroke Rehabil*. 15(5): 503-520. doi:10.1310/tsr1505-503

27. Taylor, D. M., Stone, S. D. and Huijbregts, M. P. (2012) 'Remote participants' experiences with a group-based stroke self-management program using videoconference technology', *Rural and remote health*, 12, p. 1947.

28. Mullis, R. et al. (2019) 'Improving Primary Care After Stroke (IPCAS) trial: protocol of a randomised controlled trial to evaluate a novel model of care for stroke survivors living in the community', Brit Med J Open, 9(8), p. E030285. DOI: 10.1016/j.pec.2010.07.046

29. Johnson, V. L., Apps, L., Hadjiconstantinou, M., Carey, M. E., Kreit E., Mullis, R., Mant, J., & Davies., M (2022) The development of a self-management intervention for stroke survivors - My Life After Stroke (MLAS), *Disability and Rehabilitation,* https://doi.org/10.1080/09638288.2022.2029959

30. Johnson, V. L., Apps, L., Kreit E., Mullis, R., Mant, J., & Davies., M on behalf of the MLAS development group (2022) The feasibility of a self-management programme (My Life After Stroke) for stroke survivors Disability and Rehabilitation doi.org/10.1080/09638288.2022.2029960

31. Aquino, M. R. J., Mullis, R., Kreit, E., Johnson, V., Grant, J., Lim, L., … & Mant, J. (2020). *Improving Primary Care After Stroke (IPCAS) randomised controlled trial: protocol for a multidimensional process evaluation.* Brit Med J open, 10(7), e036879. DOI: 10.1016/j.pec.2010.07.046

32. Ory, M,G., Jordan, P.J., Bazzarre, T. (2002) ‘The Behaviour Change Consortium: setting the stage for a new century of health behaviour-change research’ *Health Educ Res.* 17(5):500-511. doi:10.1093/her/17.5.500

33. Braun V, Clarke V. (2013) Thematic Analysis. American Psychological Association.

34. Forster, A. et al. (2018) ‘An intervention to support stroke survivors and their carers in the longer term (LoTS2Care): study protocol for a cluster randomised controlled feasibility trial’, Trials. BioMed Central, 19(1), pp. 1–14. DOI: 10.1186/s13063-018-2669-5

35. Stafford, M., et al. (2018) *"Briefing: Understanding the health care needs of people with multiple health conditions"* The Health Foundation, London.

36. Aquino, M. R. J. V., Turner, G. M., & Mant, J. (2019). Does characterising patterns of multimorbidity in stroke matter for developing collaborative care approaches in primary care?. *Primary Health Care Research & Development*, 20. doi:10.1017/S1463423619000240

37. Kim, J. I., Lee, S., & Kim, J. H. (2013). Effects of a web-based stroke education program on recurrence prevention behaviours among stroke patients: a pilot study. *Health education research*, *28*(3), 488-501. DOI: 10.1093/her/cyt044

For peer review only

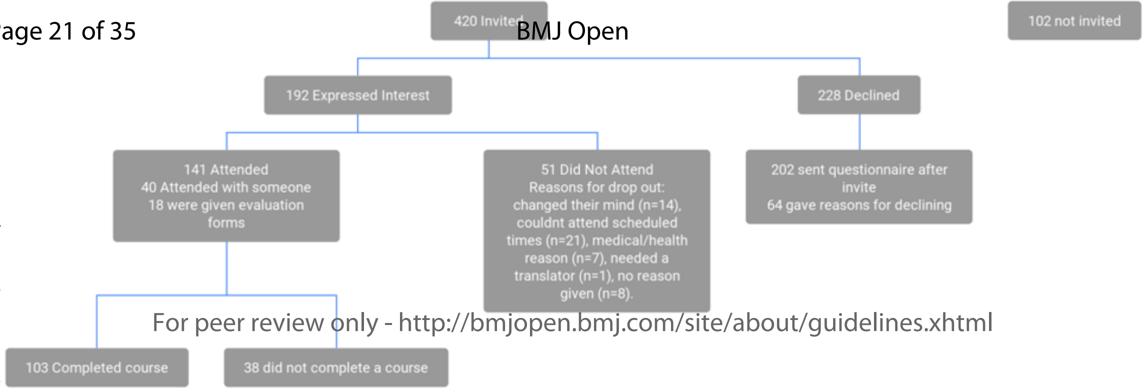

For peer review only - http://bmjopen.bmj.com/site/about/guidelines.xhtml

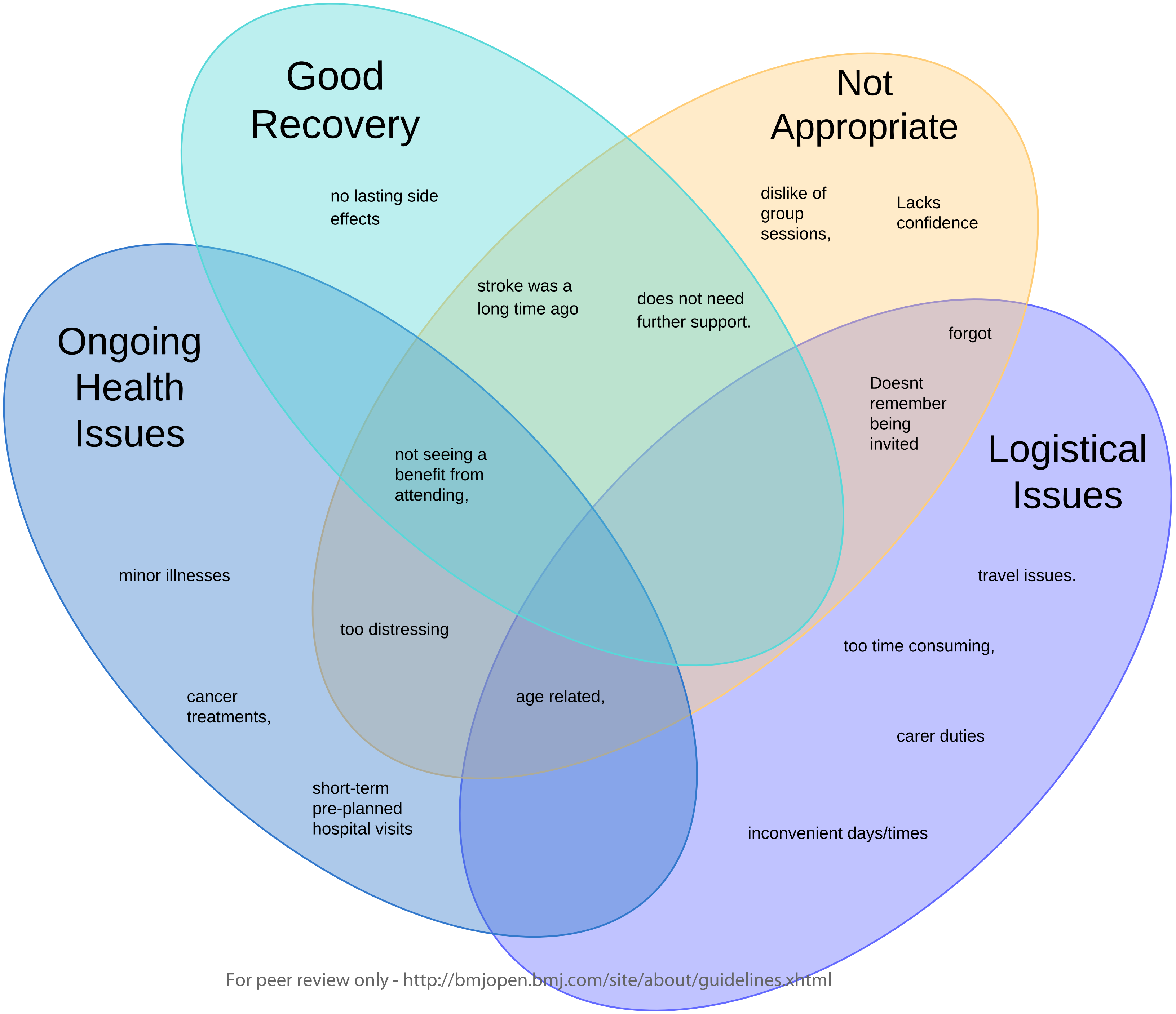

For peer review only - http://bmjopen.bmj.com/site/about/guidelines.xhtml

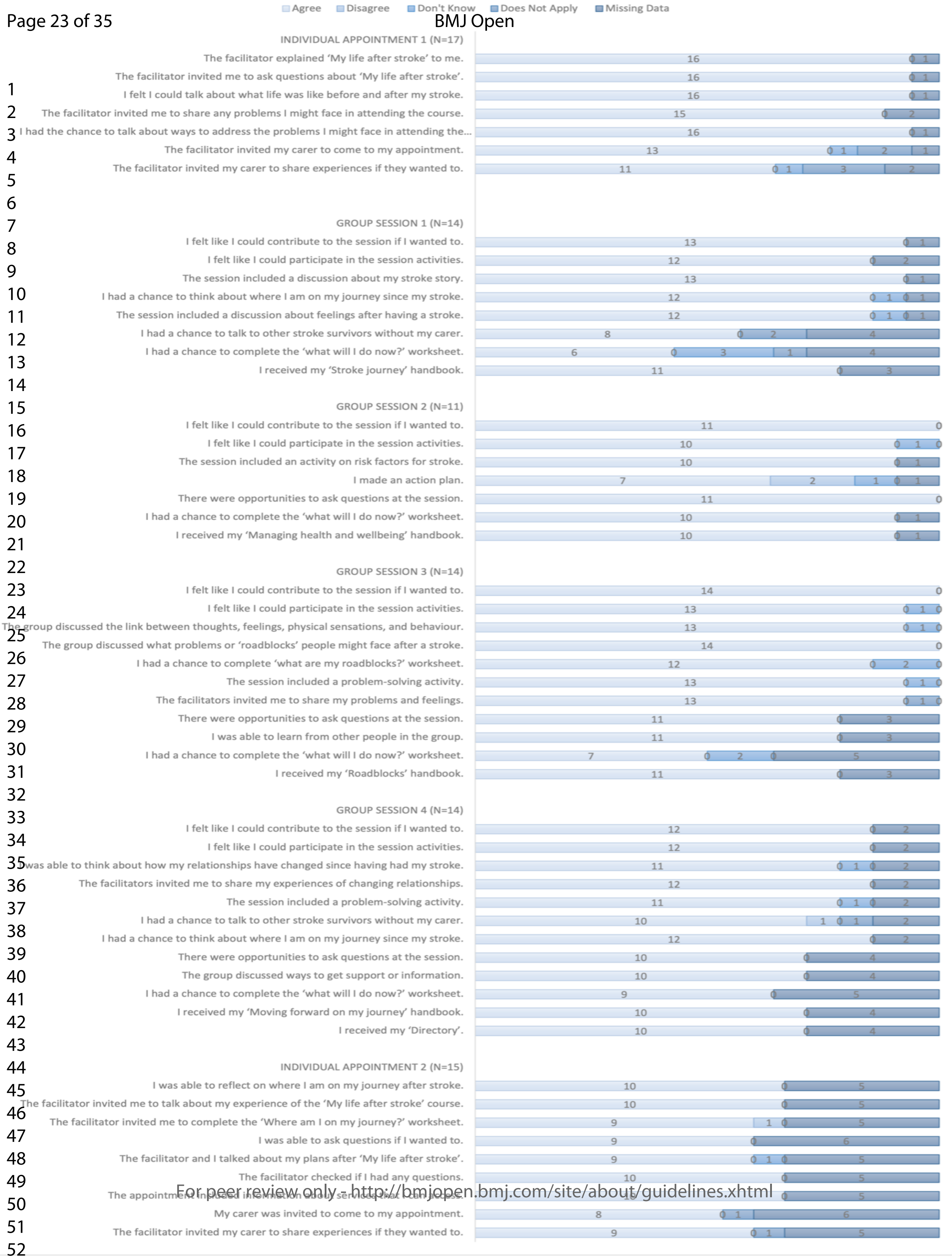
Agree
Disagree
Don't Know
Does Not Apply
Missing Data
INDIVIDUAL APPOINTMENT 1 (N=17)
The facilitator explained 'My life after stroke' to me. 16 0 1
The facilitator invited me to ask questions about 'My life after stroke'. 16 0 1
I felt I could talk about what life was like before and after my stroke. 16 0 1
The facilitator invited me to share any problems I might face in attending the course. 15 0 2
I had the chance to talk about ways to address the problems I might face in attending the… 16 0 1
The facilitator invited my carer to come to my appointment. 13 0 1 2 1
The facilitator invited my carer to share experiences if they wanted to. 11 0 1 3 2
GROUP SESSION 1 (N=14)
I felt like I could contribute to the session if I wanted to. 13 0 1
I felt like I could participate in the session activities. 12 0 2
The session included a discussion about my stroke story. 13 0 1
I had a chance to think about where I am on my journey since my stroke. 12 0 1 0 1
The session included a discussion about feelings after having a stroke. 12 0 1 0 1
I had a chance to talk to other stroke survivors without my carer. 8 0 2 4
I had a chance to complete the 'what will I do now?' worksheet. 6 0 3 1 4
I received my 'Stroke journey' handbook. 11 0 3
GROUP SESSION 2 (N=11)
I felt like I could contribute to the session if I wanted to. 11 0
I felt like I could participate in the session activities. 10 0 1 0
The session included an activity on risk factors for stroke. 10 0 1
I made an action plan. 7 2 1 0 1
There were opportunities to ask questions at the session. 11 0
I had a chance to complete the 'what will I do now?' worksheet. 10 0 1
I received my 'Managing health and wellbeing' handbook. 10 0 1
GROUP SESSION 3 (N=14)
I felt like I could contribute to the session if I wanted to. 14 0
I felt like I could participate in the session activities. 13 0 1 0
The group discussed the link between thoughts, feelings, physical sensations, and behaviour. 13 0 1 0
The group discussed what problems or 'roadblocks' people might face after a stroke. 14 0
I had a chance to complete 'what are my roadblocks?' worksheet. 12 0 2 0
The session included a problem-solving activity. 13 0 1 0
The facilitators invited me to share my problems and feelings. 13 0 1 0
There were opportunities to ask questions at the session. 11 0 3
I was able to learn from other people in the group. 11 0 3
I had a chance to complete the 'what will I do now?' worksheet. 7 0 2 0 5
I received my 'Roadblocks' handbook. 11 0 3
GROUP SESSION 4 (N=14)
I felt like I could contribute to the session if I wanted to. 12 0 2
I felt like I could participate in the session activities. 12 0 2
I was able to think about how my relationships have changed since having had my stroke. 11 0 1 0 2
The facilitators invited me to share my experiences of changing relationships. 12 0 2
The session included a problem-solving activity. 11 0 1 0 2
I had a chance to talk to other stroke survivors without my carer. 10 1 0 1 2
I had a chance to think about where I am on my journey since my stroke. 12 0 2
There were opportunities to ask questions at the session. 10 0 4
The group discussed ways to get support or information. 10 0 4
I had a chance to complete the 'what will I do now?' worksheet. 9 0 5
I received my 'Moving forward on my journey' handbook. 10 0 4
I received my 'Directory'. 10 0 4
INDIVIDUAL APPOINTMENT 2 (N=15)
I was able to reflect on where I am on my journey after stroke. 10 0 5
The facilitator invited me to talk about my experience of the 'My life after stroke' course. 10 0 5
The facilitator invited me to complete the 'Where am I on my journey?' worksheet. 9 1 0 5
I was able to ask questions if I wanted to. 9 0 6
The facilitator and I talked about my plans after 'My life after stroke'. 9 0 1 0 5
The facilitator checked if I had any questions. 10 0 5
The appointment included information about services that I can access. 10 0 5
My carer was invited to come to my appointment. 8 0 1 6
The facilitator invited my carer to share experiences if they wanted to. 9 0 1 5

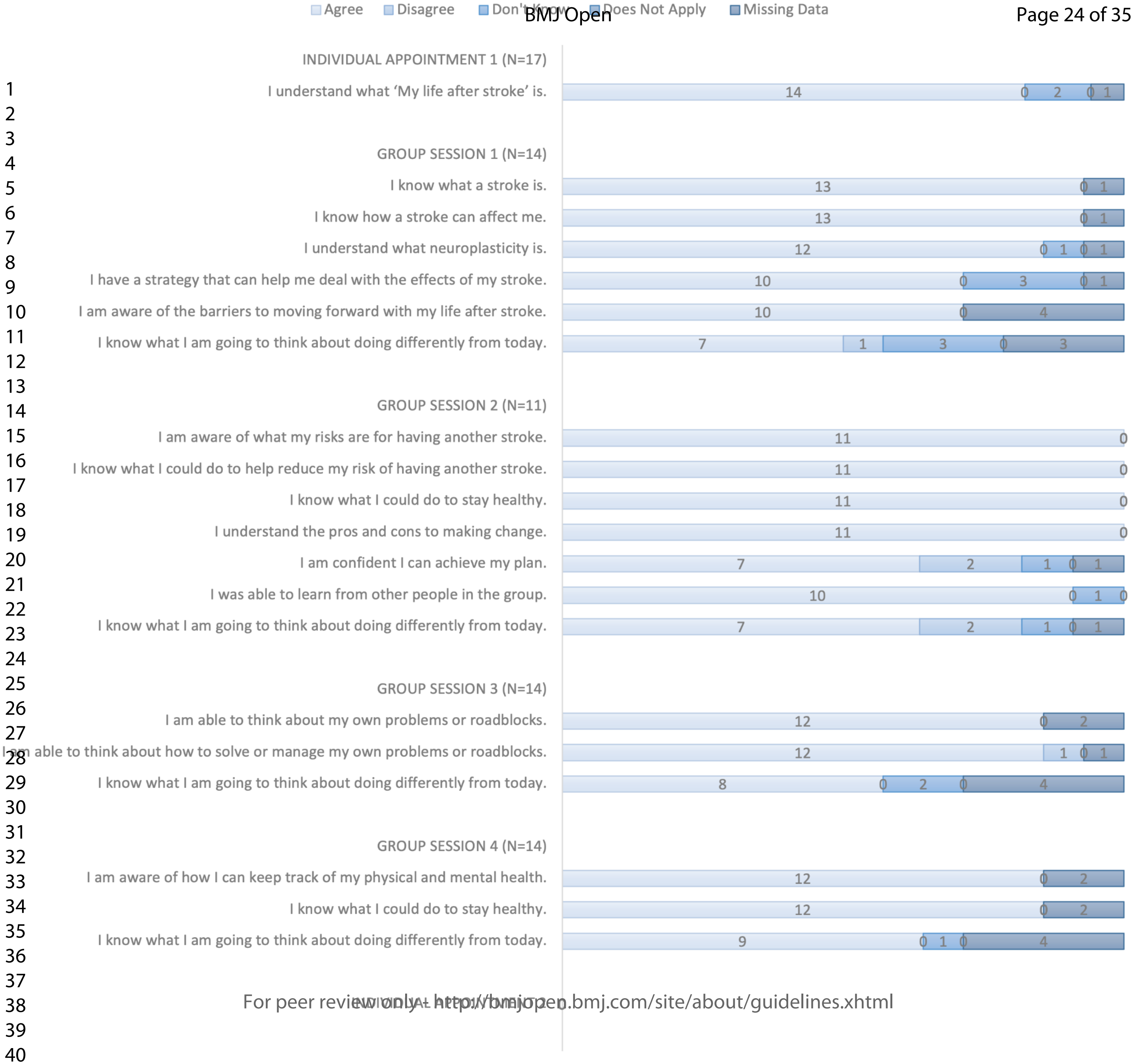
Agree
Disagree
Don't Know
Does Not Apply
Missing Data
INDIVIDUAL APPOINTMENT 1 (N=17)
I understand what 'My life after stroke' is.
GROUP SESSION 1 (N=14)
I know what a stroke is.
I know how a stroke can affect me.
I understand what neuroplasticity is.
I have a strategy that can help me deal with the effects of my stroke.
I am aware of the barriers to moving forward with my life after stroke.
I know what I am going to think about doing differently from today.
GROUP SESSION 2 (N=11)
I am aware of what my risks are for having another stroke.
I know what I could do to help reduce my risk of having another stroke.
I know what I could do to stay healthy.
I understand the pros and cons to making change.
I am confident I can achieve my plan.
I was able to learn from other people in the group.
I know what I am going to think about doing differently from today.
GROUP SESSION 3 (N=14)
I am able to think about my own problems or roadblocks.
I am able to think about how to solve or manage my own problems or roadblocks.
I know what I am going to think about doing differently from today.
GROUP SESSION 4 (N=14)
I am aware of how I can keep track of my physical and mental health.
I know what I could do to stay healthy.
I know what I am going to think about doing differently from today.

As part of the IPCAS trial you were invited by your GP or Practice Nurse to take part in the **My Life After Stroke (MLAS)** programme.

If you decided **not** to take part, please can you tell us why?

___

___

___

___

___

For peer review only - http://bmjopen.bmj.com/site/about/guidelines.xhtml

Thank you for attending **Stroke Journey**.

Please tick (✓) **agree, disagree, don't know** or **does not apply** for each of the statements below.

	Agree	Disagree	Don't know	Does not apply
1. I felt like I could contribute to the session if I wanted to.				
2. I felt like I could participate in the session activities.				
3. The session included a discussion about my **stroke story**.				
4. I had a chance to think about where I am on my **journey** since my stroke.				
5. I know what a stroke is.				
6. I know how a stroke can affect me.				
7. I understand what **neuroplasticity** is.				
8. I have a **strategy** that can help me deal with the effects of my stroke.				
9. The session included a discussion about **feelings** after having a stroke.				

Please turn over.

Improving primary care after stroke (IPCAS): A cluster randomised controlled trial
MLAS Patient Receipt Checklist – Group 1
IRAS Number: 233891
v.1.0 21-06-18

Please tick ✓ **agree**, **disagree**, **don't know** or **does not apply** for each of the statements below.

	Agree	Disagree	Don't know	Does not apply
10. I am aware of the **barriers** to moving forward with my life after stroke.				
11. I had a chance to talk to other stroke survivors **without my carer**.				
12. I had a chance to complete the **'what will I do now?'** worksheet.				
13. I know what I am going to think about doing differently from today.				
14. I received my **'Stroke journey'** handbook.				

Please write your **comments** below:

Improving primary care after stroke (IPCAS): A cluster randomised controlled trial
MLAS Patient Receipt Checklist – Group 1
IRAS Number: 233891
v.1.0 21-06-18

Thank you for attending **Managing health and wellbeing**.

Please tick (✓) **agree, disagree, don't know** or **does not apply** for each of the statements below.

	Agree	Disagree	Don't know	Does not apply
1. I felt like I could contribute to the session if I wanted to.				
2. I felt like I could participate in the session activities.				
3. The session included an activity on **risk factors for stroke**.				
4. I am aware of what my risks are for having another stroke.				
5. I know what I could do to help **reduce** my risk of having another stroke.				
6. I know what I could do to **stay healthy**.				
7. I understand the **pros and cons** to making change.				
8. I made an **action plan**.				
9. I am confident I can achieve my plan.				

Please turn over.

Please tick ✓ **agree**, **disagree**, **don't know** or **does not apply** for each of the statements below.

	Agree	Disagree	Don't know	Does not apply
10. There were opportunities to **ask questions** at the session.				
11. I was able to learn from other people in the group.				
12. I had a chance to complete the '**what will I do now?'** worksheet.				
13. I know what I am going to think about doing differently from today.				
14. I received my **'Managing health and wellbeing'** handbook.				

Please write your **comments** below:

Improving primary care after stroke (IPCAS): A cluster randomised controlled trial
MLAS Patient Receipt Checklist – Group 2
IRAS Number: 233891 v.1.0 21-06-18

Thank you for attending **Roadblocks**.

Please tick (✓) **agree, disagree, don't know** or **does not apply** for each of the statements below.

	Agree	Disagree	Don't know	Does not apply
1. I felt like I could contribute to the session if I wanted to.				
2. I felt like I could participate in the session activities.				
3. The group discussed the link between **thoughts, feelings, physical sensations, and behaviour**.				
4. The group discussed what problems or **'roadblocks'** people might face after a stroke.				
5. I had a chance to complete **'what are my roadblocks?'** worksheet.				
6. The session included a **problem-solving** activity.				
7. The facilitators invited me to share my problems and feelings.				
8. I am able to think about my own problems or roadblocks.				
9. I am able to think about how to solve or manage my own problems or roadblocks.				

Please turn over.

Improving primary care after stroke (IPCAS): A cluster randomised controlled trial
MLAS Patient Receipt Checklist – Group 3
IRAS Number: 233891
v.1.0 21-06-18

Please tick ✓ **agree**, **disagree**, **don't know** or **does not apply** for each of the statements below.

	Agree	Disagree	Don't know	Does not apply
10. There were opportunities to **ask questions** at the session.				
11. I was able to learn from other people in the group.				
12. I had a chance to complete the '**what will I do now?'** worksheet.				
13. I know what I am going to think about doing differently from today.				
14. I received my **'Roadblocks'** handbook.				

Please write your **comments** below:

Improving primary care after stroke (IPCAS): A cluster randomised controlled trial
MLAS Patient Receipt Checklist – Group 3
IRAS Number: 233891
v.1.0 21-06-18

Thank you for attending **'Moving forward on my journey'**.

Please tick (✓) **agree, disagree, don't know** or **does not apply** for each of the statements below.

	Agree	Disagree	Don't know	Does not apply
1. I felt like I could contribute to the session if I wanted to.				
2. I felt like I could participate in the session activities.				
3. I was able to think about how my relationships have changed since having had my stroke.				
4. The facilitators invited me to share my experiences of changing relationships.				
5. The session included a **problem-solving** activity.				
6. I had a chance to talk to other stroke survivors **without my carer**.				
7. I had a chance to think about where I am on my **journey** since my stroke.				
8. I am aware of how I can keep track of my **physical and mental health**.				
9. I know what I could do to **stay healthy**.				

Please turn over.

Improving primary care after stroke (IPCAS): A cluster randomised controlled trial
MLAS Patient Receipt Checklist – Group 4
IRAS Number: 233891 v.1.0 21-06-18

Please tick ✓ **agree**, **disagree**, **don't know** or **does not apply** for each of the statements below.

	Agree	Disagree	Don't know	Does not apply
10. There were opportunities to **ask questions** at the session.				
11. The group discussed ways to get **support or information**.				
12. I had a chance to complete the '**what will I do now?'** worksheet.				
13. I know what I am going to think about doing differently from today.				
14. I received my **'Moving forward on my journey'** handbook.				
15. I received my **'Directory'**.				

Please write your **comments** below:

Improving primary care after stroke (IPCAS): A cluster randomised controlled trial
MLAS Patient Receipt Checklist – Group 4
IRAS Number: 233891 v.1.0 21-06-18

Thank you for attending **Individual appointment 1**.

Please tick (✓) **agree, disagree, don't know** or **does not apply** for each of the statements below.

	Agree	Disagree	Don't know	Does not apply
1. The facilitator explained **'My life after stroke'** to me.				
2. The facilitator invited me to **ask questions** about 'My life after stroke'.				
3. I understand what **'My life after stroke'** is.				
4. I felt I could talk about what life was like **before and after** my stroke.				
5. The facilitator invited me to share any **problems I might face** in attending the course.				
6. I had the chance to talk about **ways to address** the problems I might face in attending the course.				
7. The facilitator invited my carer to come to my appointment.				
8. The facilitator invited my carer to **share experiences** if they wanted to.				

Please turn over.

Improving primary care after stroke (IPCAS): A cluster randomised controlled trial
MLAS Patient Receipt Checklist – Individual Appointment 1
IRAS Number: 233891
v.1.0 21-06-18

Please write your **comments** below:

Improving primary care after stroke (IPCAS): A cluster randomised controlled trial
MLAS Patient Receipt Checklist – Individual Appointment 1
IRAS Number: 233891
v.1.0 21-06-18

Thank you for attending **Individual appointment 2**.

Please tick (✓) **agree, disagree, don't know** or **does not apply** for each of the statements below.

	Agree	Disagree	Don't know	Does not apply
1. I was able to reflect on where I am on my **journey** after stroke.				
2. The facilitator invited me to talk about my experience of the **'My life after stroke'** course.				
3. The facilitator invited me to complete the **'Where am I on my journey?'** worksheet.				
4. I was able to **ask questions** if I wanted to.				
5. The facilitator and I talked about my **plans** after 'My life after stroke'.				
6. The facilitator checked if I had any questions.				
7. The appointment included **information about services** that I can access.				

Please turn over.

Please tick ✓ **agree**, **disagree**, **don't know** or **does not apply** for each of the statements below.

	Agree	Disagree	Don't know	Does not apply
8. My carer was invited to come to my appointment.				
9. The facilitator invited my carer to **share experiences** if they wanted to.				

Please write your **comments** below:

Improving Primary Care After Stroke: A randomised controlled trial to evaluate a new model of care for stroke survivors living in the community

Proposed survivor Topic Guide for semi-structured interviews

Interview with a stroke survivor – Intervention Arm

Thank you for agreeing to let me come and talk with you. We're interested to hear from people who've taken part in the IPCAS trial, and your experiences of stroke care so far.

Confidentiality & anonymity. Tape recording. Stopping / Pausing. Anything you don't want to talk about. I'm not a clinician. Any questions?

Is there anything you'd like me to know before we start that would make things easier for you? For instance, do you want me to sit one side or another? Do you sometimes find it difficult to find the right word? Or to understand what someone's saying? Is there anything I can do to make things better for you?

Do you have any questions?
Provide brief outline / reminder of what the study was about

Review at GP Surgery

Can you tell me about your experience of the stroke review you attended at your GP surgery?
(*prompts: date of review, person who did the review, checklist*)

Can you tell me about what happened at the review?
(*prompts: checklist discussion, action plan, blood pressure, any recommendations regarding social care/support services*)

What were your expectations of the stroke review?
(prompts: were they met/not met? How?)

Overall, what did you think about the checklist used for your review?
For example, was it helpful/useful? Why/why not?
(*prompts: did it help you identify and discuss needs you may have, were you referred on, were you given information about services available to you*)

Overall, what did you think about the review?
For example, was it helpful/useful? Why/why not?
(*prompts: were you referred on, did you attend MLAS, were you given information about services available to you*)

How did this review meet your needs in relation to your stroke?
Is there anything else you wanted to share about your stroke review today?

If no review – do you think a review of your care needs at your GP Surgery would have been helpful/useful? Why/why not?

For peer review only - http://bmjopen.bmj.com/site/about/guidelines.xhtml

MLAS self-management course

Can you tell me a little bit about your experience of the MLAS self-management course?
(prompts: Did you attend all sessions?; What parts of MLAS did you like the most/least liked? Why?)

How did you hear about MLAS?
(*prompts: what information did you get?*)

How did MLAS meet your needs in relation to your stroke?

Overall, what did you think about the MLAS course?
(*prompts: did it help you to feel more confident/independent/empowered to manage you stroke related needs*)

Was it helpful/useful? If yes, how? If no, why not?

Is there anything else you wanted to share about participating in MLAS today?

If participant did not attend MLAS:
Were you told about MLAS at the review? If yes: can you please tell me a bit about your reasons for not attending MLAS (prompts as below).
Did you choose not to attend, or were there other reasons (e.g. logistic, travel, cost, time) that prevented you from attending?

Direct Point of Contact

Tell me about how you used the direct point of contact service that you were told about at your stroke review.

If participant did not use the service, explore: what were your reasons for not using the direct point of contact?
(prompts: no information given, did not want to bother the Practice staff, not feel it was worth it, couldn't get through on the telephone)

Would you have liked to be able to contact someone at your GP surgery about your stroke needs?

How useful do you think it would have been to have a direct point of contact/somebody specific to call? Explore reasons why/why not.

What prompted you to call the direct point of contact service?

How much did you use the direct point of contact service?

How useful was this direct point of contact service?
(prompts: What kind of information/advice/support did you receive?)

What do you think about the response you received?
(prompts: Called back in a timely manner, extent to which issues/questions/needs were addressed)

For peer review only - http://bmjopen.bmj.com/site/about/guidelines.xhtml

Overall

Since you entered the IPCAS trial, what changes have you noticed in how your GP surgery deals with your needs/concerns/issues?

(*prompts: easier/harder to contact, discuss problems, more/less helpful service*)

What are your views about the care that you received as a whole?

(prompts: Thoughts on the information/advice given throughout? What did you learn (if anything)? How are you using your learning?)

Is there anything you think needs to change about your stroke care? If so, what are these changes, and why?

Is there anything else you would like to discuss relating to the care you received as part of the IPCAS trial?

Thank you very much. These are all questions I had. Is there anything you would like to ask?

Thank you very much for your time.

For peer review only

For peer review only - http://bmjopen.bmj.com/site/about/guidelines.xhtml

CONSORT 2010 checklist of information to include when reporting a randomised trial*

Section/Topic	Item No	Checklist item	Reported on page No
**Title and abstract**			
	1a	Identification as a randomised trial in the title	1
	1b	Structured summary of trial design, methods, results, and conclusions (for specific guidance see CONSORT for abstracts)	2
**Introduction**			
Background and objectives	2a	Scientific background and explanation of rationale	4
	2b	Specific objectives or hypotheses	4
**Methods**			
Trial design	3a	Description of trial design (such as parallel, factorial) including allocation ratio	5
	3b	Important changes to methods after trial commencement (such as eligibility criteria), with reasons	5
Participants	4a	Eligibility criteria for participants	5
	4b	Settings and locations where the data were collected	5
Interventions	5	The interventions for each group with sufficient details to allow replication, including how and when they were actually administered	5
Outcomes	6a	Completely defined pre-specified primary and secondary outcome measures, including how and when they were assessed	5,6
	6b	Any changes to trial outcomes after the trial commenced, with reasons	5,6
Sample size	7a	How sample size was determined	5,6
	7b	When applicable, explanation of any interim analyses and stopping guidelines	5,6
Randomisation:			
Sequence generation	8a	Method used to generate the random allocation sequence	5,6
	8b	Type of randomisation; details of any restriction (such as blocking and block size)	5,6
Allocation concealment mechanism	9	Mechanism used to implement the random allocation sequence (such as sequentially numbered containers), describing any steps taken to conceal the sequence until interventions were assigned	5,6
Implementation	10	Who generated the random allocation sequence, who enrolled participants, and who assigned participants to interventions	5,6
Blinding	11a	If done, who was blinded after assignment to interventions (for example, participants, care providers, those	n/a

For peer review only

		assessing outcomes) and how	
	11b	If relevant, description of the similarity of interventions	5,6
Statistical methods	12a	Statistical methods used to compare groups for primary and secondary outcomes	5,6
	12b	Methods for additional analyses, such as subgroup analyses and adjusted analyses	5,6
**Results**			
Participant flow (a diagram is strongly recommended)	13a	For each group, the numbers of participants who were randomly assigned, received intended treatment, and were analysed for the primary outcome	7
	13b	For each group, losses and exclusions after randomisation, together with reasons	7
Recruitment	14a	Dates defining the periods of recruitment and follow-up	7
	14b	Why the trial ended or was stopped	7
Baseline data	15	A table showing baseline demographic and clinical characteristics for each group	7
Numbers analysed	16	For each group, number of participants (denominator) included in each analysis and whether the analysis was by original assigned groups	7
Outcomes and estimation	17a	For each primary and secondary outcome, results for each group, and the estimated effect size and its precision (such as 95% confidence interval)	7
	17b	For binary outcomes, presentation of both absolute and relative effect sizes is recommended	7
Ancillary analyses	18	Results of any other analyses performed, including subgroup analyses and adjusted analyses, distinguishing pre-specified from exploratory	7,8,9,10,11,12,13
Harms	19	All important harms or unintended effects in each group (for specific guidance see CONSORT for harms)	
**Discussion**			
Limitations	20	Trial limitations, addressing sources of potential bias, imprecision, and, if relevant, multiplicity of analyses	13
Generalisability	21	Generalisability (external validity, applicability) of the trial findings	13
Interpretation	22	Interpretation consistent with results, balancing benefits and harms, and considering other relevant evidence	13
**Other information**			
Registration	23	Registration number and name of trial registry	16
Protocol	24	Where the full trial protocol can be accessed, if available	16
Funding	25	Sources of funding and other support (such as supply of drugs), role of funders	16

*We strongly recommend reading this statement in conjunction with the CONSORT 2010 Explanation and Elaboration for important clarifications on all the items. If relevant, we also recommend reading CONSORT extensions for cluster randomised trials, non-inferiority and equivalence trials, non-pharmacological treatments, herbal interventions, and pragmatic trials. Additional extensions are forthcoming: for those and for up to date references relevant to this checklist, see www.consort-statement.org.

For peer review only - http://bmjopen.bmj.com/site/about/guidelines.xhtml
